# The landscape of lncRNAs in *Cydia pomonella* provides insights into their signatures and potential roles in transcriptional regulation

**DOI:** 10.1186/s12864-020-07313-3

**Published:** 2021-01-05

**Authors:** Longsheng Xing, Yu Xi, Xi Qiao, Cong Huang, Qiang Wu, Nianwan Yang, Jianyang Guo, Wanxue Liu, Wei Fan, Fanghao Wan, Wanqiang Qian

**Affiliations:** 1 Shenzhen Branch, Guangdong Laboratory for Lingnan Modern Agriculture, Genome Analysis Laboratory of the Ministry of Agriculture, Agricultural Genomics Institute at Shenzhen, Chinese Academy of Agricultural Sciences, Shenzhen, 518120 China; 2 State Key Laboratory for Biology of Plant Diseases and Insect Pests, Institute of Plant Protection, Chinese Academy of Agricultural Sciences, Beijing, 100193 China

**Keywords:** Long noncoding RNA, Conservation, Synteny, Transcriptional regulation, *Cydia pomonella*

## Abstract

**Background:**

Long noncoding RNAs (lncRNAs) have emerged as an important class of transcriptional regulators in cellular processes. The past decades have witnessed great progress in lncRNA studies in a variety of organisms. The codling moth (*Cydia pomonella* L.) is an important invasive insect in China. However, the functional impact of lncRNAs in this insect remains unclear. In this study, an atlas of codling moth lncRNAs was constructed based on publicly available RNA-seq datasets.

**Results:**

In total, 9875 lncRNA transcripts encoded by 9161 loci were identified in the codling moth. As expected, the lncRNAs exhibited shorter transcript lengths, lower GC contents, and lower expression levels than protein-coding genes (PCGs). Additionally, the lncRNAs were more likely to show tissue-specific expression patterns than PCGs. Interestingly, a substantial fraction of the lncRNAs showed a testis-biased expression pattern. Additionally, conservation analysis indicated that lncRNA sequences were weakly conserved across insect species, though additional lncRNAs with homologous relationships could be identified based on synteny, suggesting that synteny could be a more reliable approach for the cross-species comparison of lncRNAs. Furthermore, the correlation analysis of lncRNAs with neighbouring PCGs indicated a stronger correlation between them, suggesting potential *cis*-acting roles of these lncRNAs in the regulation of gene expression.

**Conclusions:**

Taken together, our work provides a valuable resource for the comparative and functional study of lncRNAs, which will facilitate the understanding of their mechanistic roles in transcriptional regulation.

## Background

Over the past decade, long noncoding RNAs (lncRNAs) have been recognized as important regulatory factors involved in a wide range of physiological processes, such as cell differentiation [1], development [2], X-chromosome inactivation [3], immune responses [4] and human diseases [5]. Due to lack of a scientific definition, lncRNAs are conventionally defined as a class of non-coding RNAs with a size > 200 nt that are devoid of open reading frames [6]. Previous studies have demonstrated that lncRNAs are distributed in almost all living organisms, including humans, animals, plants, nematodes, yeast, bacteria and viruses [7]. Compared with mRNAs, lncRNAs usually exhibit a lower GC content, poorer sequence conservation and lower expression levels. According to the genomic context of lncRNAs, they are divided into four subclasses: intergenic, intronic, sense, and antisense lncRNAs [8]. Alternatively, lncRNAs are classified as *cis*- or *trans*-lncRNAs, which is mainly dependent on whether the expression of neighbouring or distant target protein-coding genes (PCGs) is regulated [8]. Due to their larger size and more complex secondary structure, the mechanisms of action of lncRNAs are highly diversified. The regulation of lncRNAs can encompass multi-layered activities, mainly at the pre-transcriptional, transcriptional and post-transcriptional levels. Generally, lncRNAs can serve as chromatin modifiers, RNA decoys, transcriptional coactivators, ribonucleoprotein components, and microRNA sponges to regulate the expression of target genes [9]. Given the critical roles of lncRNAs in cellular processes, many efforts have been made to identify and characterize the landscape of lncRNAs in many organisms.

Previous studies and a series of databases have been devoted to cataloguing lncRNAs from model organisms, which were limited to humans or mice. Recently, an increasing number of studies have reported the identification of lncRNAs in insects [10–17]. In the model insect *Drosophila melanogaster*, the identification and evolutionary analysis of lncRNA loci have been performed on a genome-wide scale, revealing developmental expression profiles and potential functional analogues in mammals [11]. Jenkins et al. employed deep RNA sequencing to systematically identify lncRNAs across the genus *Anopheles* and revealed conserved secondary structures [12]. Etebari et al. identified lncRNAs in the *Aedes aegypti* genome and revealed their association with *Wolbachia* and dengue virus infection [13]. In honey bees, genome-wide analysis identified 2470 and 1514 lncRNAs in *Apis cerana* and *Apis mellifera*, respectively. Intriguingly, 10 lncRNAs were demonstrated to play roles during the viral infection of honey bees [15]. In the silkworm, a total of 11,810 lncRNAs derived from 5556 loci were identified and characterized based on strand-specific and poly(A)-enriched RNA-seq data [16]. In the diamondback moth, genome-wide lncRNAs were identified and found to show differential expression in insecticide resistant strains [18]. In addition to the widespread discovery of lncRNAs, progress has also been made in the functional study of lncRNAs in insects. For example, a male-specific lncRNA was found to play an important role in accessory gland development and male fertility in *Drosophila* [19]. Additionally, CRISPR/Cas9-based knockdown demonstrated that dozens of testis-specific *Drosophila* lncRNAs play critical roles in spermatogenesis [20]. Valanne et al. reported that an immune-inducible lncRNA was involved in the regulation of immunity and metabolism in *Drosophila* [21]. More recently, Zhang et al. found that the *Drosophila* lncRNA VINR could coordinate host antiviral immunity by activating noncanonical innate immune signalling [22].

The codling moth (*Cydia pomonella* L.), which belongs to the Tortricidae family (Lepidoptera), is one of the most harmful invasive insect species in China [23]. It can infest dozens of host plants, particularly among pome fruits and walnuts. The first report of codling moths was not published until 1953 in Xinjiang [24]. To date, the distribution of codling moths has expanded to 131 counties in seven provinces in China [25]. Moreover, the development of domestic and international trade, transportation and tourism has accelerated its spread to other places, posing a great threat to the production of apples in China, which is a major apple-growing region of the world [23]. It has been reported that the codling moth can cause estimated economic losses of as much as $605 million per year in China [25]. During recent decades, most studies have mainly focused on the chemical ecology and insecticide resistance of codling moths [26, 27]. However, the roles of non-coding RNAs in this insect remain poorly understood. In our previous study, we reported a chromosome-level assembly of the *C. pomonella* genome [28]. In the present study, we employed publicly available RNA-seq data to obtain a comprehensive landscape of lncRNAs in this insect. To the best of our knowledge, this is the first study aimed at the systematic identification and characterization of lncRNAs in an invasive insect. Our study will benefit future in-depth investigations of lncRNAs in the codling moth, thus facilitating the dissection of the transcriptional regulation of lncRNAs in other invasive insects.

## Results

### Identification of 9875 lncRNAs in the genome of *C. pomonella*

To systematically identify lncRNA transcripts from the *C. pomonella* genome, we used RNA-seq datasets generated from nine distinct tissues and five different developmental stages (Table S1). A total of 21 RNA-seq libraries encompassing 0.99 billion paired-end reads were utilized for lncRNA identification in this study. A flowchart of the lncRNA identification procedure is shown in Fig. S1. Briefly, the transcriptome was reconstructed via GSNAP mapping and StringTie assembly. Subsequently, gffcompare was used for the classification of these merged transcripts. Only novel transcripts were retained for further analysis based on their class codes (“u”, “i”, “x”). Then, a series of filtering strategies were employed to rule out transcripts with coding potential, yielding 9875 candidate lncRNAs encoded by 9161 loci.

**Fig. 1:**
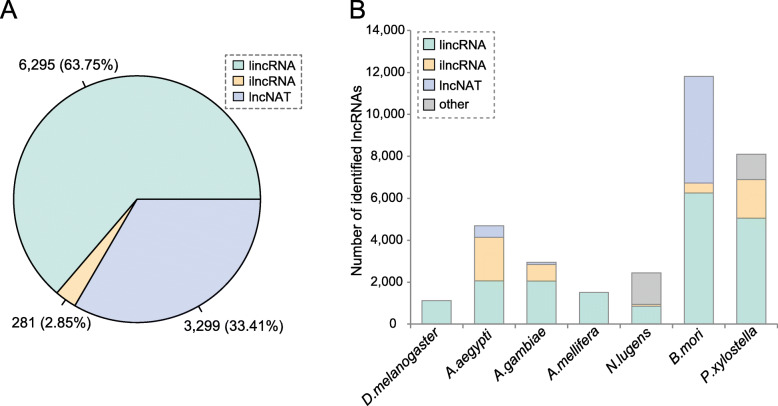
Classification of codling moth lncRNAs and summary of lncRNAs reported in other insect species. **a** Pie chart displaying the composition of three classes of lncRNAs: long intergenic noncoding RNAs (lincRNA), intronic long noncoding RNAs (ilncRNA) and long noncoding natural antisense transcripts (lncNAT). Notably, lincRNAs represent the most abundant subclass of lncRNAs in *C. pomonella*, followed by lncNATs. **b** Bar plot representation of lncRNAs reported in seven other insect species. For *D. melanogaster* and *A. mellifera*, only lincRNAs were reported in the corresponding literature. For *A. aegypti*, only novel lncRNAs reported in the literature (lncRNAs in genome annotation not included here) are shown in the plot. For simplicity, the non-intergenic and non-intronic lncRNAs from *N. lugens* and *P. xylostella* were assigned to the other category, due to the different classification types adopted in the literature. Abbreviations: *D. melanogaster*, *Drosophila melanogaster*; *A. aegypti*, *Aedes aegypti*; *A. gambiae*, *Anopheles gambiae*; *A. mellifera*, *Apis mellifera*; *N. lugens*, *Nilaparvata lugens*; *B. mori*, *Bombyx mori*; *P. xylostella*, *Plutella xylostella*

According to their class codes, these lncRNAs were categorized into three subclasses: long intergenic noncoding RNAs (lincRNA, “u”), intronic long noncoding RNAs (ilncRNA, “i”) and long noncoding natural antisense transcripts (lncNAT, “x”). No sense lncRNAs were identified in our analysis because transcripts overlapping PCG exons on the same strand were not considered. As shown in the pie chart (Fig. 1a), the majority of lncRNAs (6295, 63.75%) were located in intergenic regions of the genome. More than one-third of lncRNAs (3299, 33.41%) were annotated as originating from the antisense transcripts, while a minority of lncRNAs (281, 2.85%) were found in intronic regions of the genome. For comparison, we simply summarized the number of lncRNAs reported in seven other insect species previously [11, 12, 14–18]. As shown in Fig. 1b, the number of lncRNAs varied greatly in different species. Overall, lincRNAs were dominant among the three classes of lncRNAs in most species, while the percentages of ilncRNAs and lncNATs varied among different insect species. Statistical test showed that the distribution pattern of the three classes of lncRNAs was significantly different among three Lepidoptera insects (χ^2^ = 3803.6, df = 4, *P* < 2.2e-16, Chi-squared test) (Fig. S2A). As a control, we also identified lncRNAs in two other Lepidoptera insects (*Bombyx mori* and *Plutella xylostella*) using our pipeline. The results showed that 2531 (1311 lincRNAs, 30 ilncRNAs, and 1190 lncNATs) and 2198 (1024 lincRNAs, 178 ilncRNAs, and 996 lncNATs) lncRNA transcripts were identified in *B. mori* and *P. xylostella*, respectively (Fig. S2B). The genomic positions of the three classes of lncRNAs in codling moth, *B. mori*, and *P. xylostella* identified by our pipeline are provided in annotation files in GTF format (Table S2, 3, 4). In the remainder of this paper, we used the lncRNA dataset reported in the literature for further analysis.

To inspect the distribution of lncRNAs across different chromosomes, we generated a circular plot to obtain a straightforward view (Fig. S3A). The number of lncRNAs distributed on each chromosome is listed in Table S5, showing that the majority of lncRNAs (9721, 98.44%) could be anchored onto chromosomes. Additionally, the number of lncRNAs on each chromosome showed a strong positive correlation with chromosome size (R = 0.921, *P* = 1.396e-12, Pearson correlation coefficient [PCC]) (Fig. S3B). Similarly, the number of lncRNAs was highly correlated with that of PCGs on each chromosome (R = 0.937, *P* = 6.792e-14, PCC) (Fig. S3C). As shown in the sequence logo, the pattern surrounding the splice sites of the lncRNAs was almost the same as for the mRNAs (Fig. S4). The only difference was that alternative splicing signals exist in the lncRNAs in addition to the canonical GT/AG splicing signal. Collectively, we obtained a large set of lncRNAs in *C. pomonella* and determined their distribution pattern on chromosomes.

### Genomic characteristics of lncRNAs identified in the codling moth

**Fig. 2:**
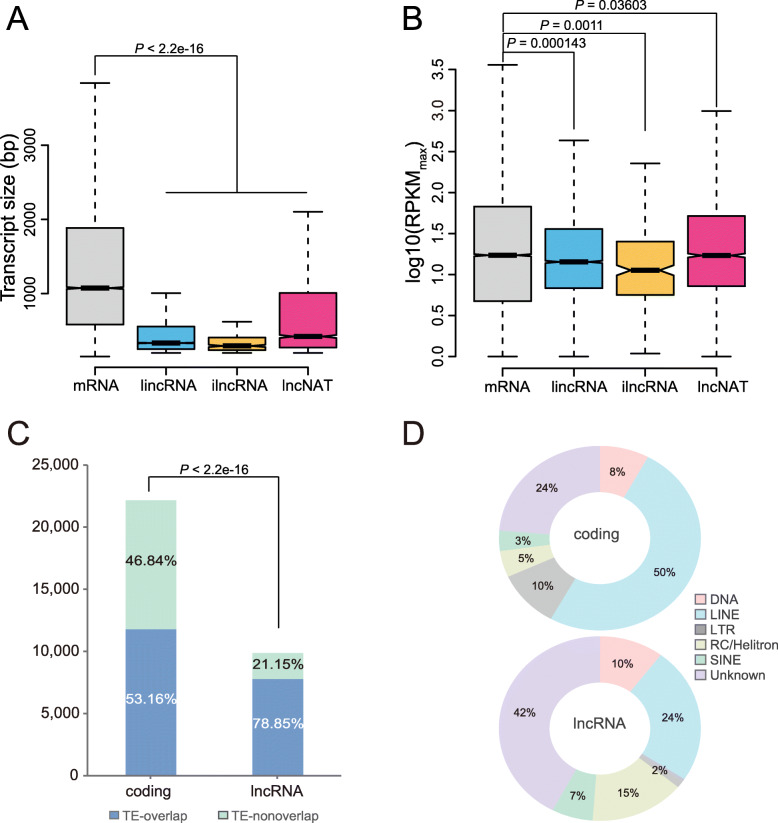
Sequence characteristics of lncRNA transcripts. **a** Box plot showing the transcript size distribution of *C. pomonella* lncRNA and mRNA transcripts. As shown in the figure, lncRNAs are significantly smaller in size than mRNAs. **b** Comparison of expression levels between mRNAs and three subclasses of lncRNAs in *C. pomonella*. The maximal RPKM values in all samples were used for comparison. As shown in the figure, the y axis was log10 scaled. The two-tailed Wilcoxon rank sum test was used for the determination of statistical significance. Relationship of TEs with PCGs and lncRNAs. **c** Stacked bar plot for the presentation of the number of PCGs and lncRNAs that overlapped with TEs. Statistical significance was determined using Fisher’s exact test. **d** Pie chart showing the distribution pattern of different classes of TEs overlapping with PCGs (top) and lncRNAs (bottom)

To explore the characteristics of the lncRNAs found in *C. pomonella*, we performed a systematic comparison of many aspects between mRNAs and lncRNAs. First, the transcript size of the lncRNAs was significantly shorter than that of the mRNAs (Fig. 2a). The median size of the mRNAs was 1074 bp, which was approximately two or three-fold that of the lncRNAs (*P* < 2.2e-16, mRNA vs. lincRNA, ilncRNA, and lncNAT, Wilcoxon rank sum test). In terms of transcript size, lncNATs were the longest class (median size = 422 bp), followed by lincRNAs (median size = 334 bp), while ilncRNAs exhibited the shortest transcript size (median size = 295 bp). The frequency of exon numbers was also analysed for each transcript type. The results showed that the vast majority of lncRNAs presented only two exons (lincRNA, 86.94%; ilncRNA, 96.80%; lncNAT, 85.30%), while mRNAs exhibited a broad distribution range of exon numbers (Fig. S5A, mRNAs vs. all lncRNAs: *P* < 2.2e-16, Wilcoxon rank sum test). To determine whether the difference in transcript size between mRNA and lncRNA was solely caused by the exon number, we performed a statistical analysis of the exon size of lncRNAs as well as mRNAs. Only a slight difference was observed in the median size of exons (162 bp for mRNA, 158 bp for lncRNA). However, statistical analysis demonstrated that the difference in exon size was significant (Fig. S5B, *P* < 2.2e-16, Wilcoxon rank sum test). By contrast, the intron size in lncRNAs (median size: 3328) was much larger than that in mRNAs (median size: 473) by approximately an order of magnitude (Fig. S5C, *P* < 2.2e-16, Wilcoxon rank sum test).

Second, we found that lncRNAs (average value: 40%) had a lower GC content than that of mRNAs (average value: 48.34%) (Fig. S5D, *P* < 2.2e-16, Wilcoxon rank sum test). It was observed that lincRNA presented a significantly higher average GC ratio than those of the other two classes (lincRNA: 38.068%, ilncRNA: 37.003%, lncNAT: 37.001%). To evaluate the expression profile of lncRNAs across different tissues and developmental stages, the maximal RPKM (Reads Per Kilobase of exon model per Million mapped reads) value of the lncRNAs across all the samples was compared with that of the mRNAs (Fig. 2b). The maximal RPKM values of the lincRNAs and ilncRNAs were significantly lower than those of the mRNAs (lincRNA vs. mRNA: *P* = 0.000143, ilncRNA vs. mRNA: *P* = 0.0011, Wilcoxon rank sum test). On the other hand, lncNATs showed a slightly higher expression level than mRNAs (*P* = 0.03603, Wilcoxon rank sum test).

Additionally, we determined the positional relationships between lncRNAs and transposable elements (TEs) in the context of the genome, and such an analysis was conducted for mRNAs as well. As depicted in Fig. 2c, a total of 11,782 PCGs were found to overlap with TEs predicted previously, accounting for 53.16% of all PCGs. By contrast, a significantly higher percentage (7786 out of 9875, 78.85%) of lncRNAs overlapped with TEs (*P* < 2.2e-16, Fisher’s exact test). Furthermore, we compared overlapping TE categories between PCGs and lncRNAs. For lncRNAs, the majority of overlapping TEs were classified as unknown. LINEs (long interspersed nuclear elements), RC/Helitrons and DNA transposons represented the top three most abundant TEs overlapped with lncRNAs. On the other hand, SINEs (short interspersed nuclear elements) and LTRs (long terminal repeat retrotransposons) were rarely associated with lncRNAs (Fig. 2d). However, the distribution pattern of TE classes was different in PCGs. Strikingly, LINEs accounted for approximately half of the TEs overlapping with PCGs, representing the most abundant category, followed by unknown and LTR-type TEs. Only a small portion of the TEs overlapping with PCGs were assigned as RC/Helitrons and SINEs.

### Spatial- and temporal-specific expression patterns of lncRNAs in the codling moth

**Fig. 3:**
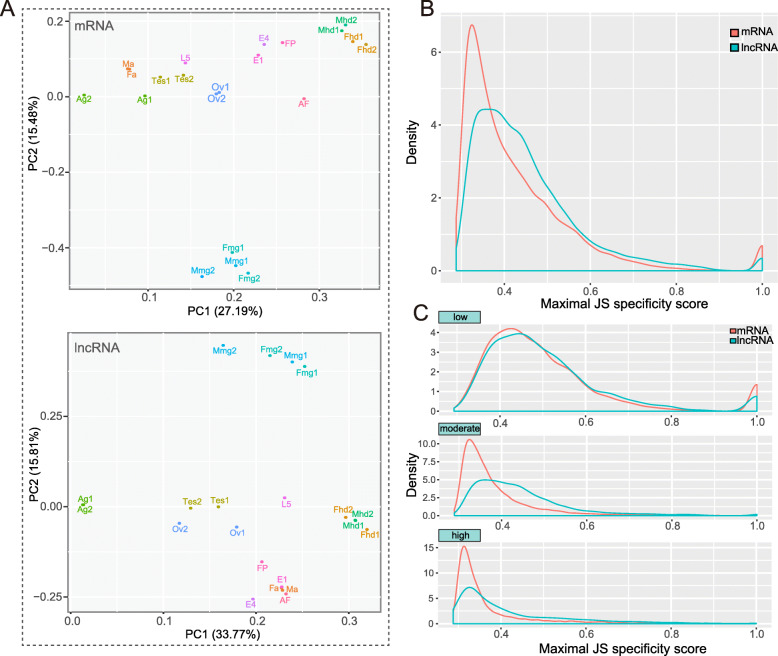
Discrete expression pattern and tissue specificity of lncRNAs in *C. pomonella*. **a** Principal component analysis (PCA) of 21 samples across multiple tissues and developmental stages based on normalized mRNA (upper) and lncRNA (lower) expression levels. Abbreviations are listed as follows: Ag, accessory gland; Ma, male antennae; Fa, female antennae; Tes, testis; Ov, ovary; Mhd, male head; Fhd, female head; Mmg, male midgut; Fmg, female midgut; E1, egg day 1; E4, egg day 4; L5, 5th-instar larva; FP, female pupa; AF, adult female. **b** Density plot showing the distribution of tissue specificity scores for all expressed PCGs and lncRNAs in *C. pomonella*. The statistical significance of the difference in tissue specificity score between lncRNAs and PCGs was demonstrated by the Kolmogorov-Smirnov test (D = 0.29563, *P* < 2.2e-16). **c** Distribution of tissue specificity scores for PCGs and lncRNAs that were assigned to the low (RPKM_max_ < 5.0), moderate (5.0 ≤ RPKM_max_ < 50.0) and high group (RPKM_max_ ≥ 50.0) based on the maximum RPKM value for gene

To explore the expression pattern of mRNAs and lncRNAs across all the samples collected in different tissues and developmental stages, we performed a principal component analysis (PCA) to distinguish these distinct sample types based on the RPKM values of coding genes and lncRNAs, respectively. To demonstrate the relationships among these samples, we employed the top two principal components to group them. The results showed that PCGs displayed a discrete expression pattern across distinct tissues (Fig. 3a, top). Basically, coding genes were expressed in a tissue-dependent manner. Almost all male and female samples for the same tissue could be clustered together. Among the samples collected at different developmental stages, embryos at day 1 and day 4, and pupa showed a similar expression pattern, while 5th-instar larva and adult females exhibited significant differences from each other. Similarly, tissue samples could be clearly separated based on the expression levels of lncRNAs (Fig. 3a, bottom). The difference was that almost all the developmental stage samples could be clustered together except for the 5th-instar larva sample. Additionally, the antenna samples were located adjacent to developmental stage samples based on the RPKM values of the lncRNAs. In contrast, the antenna samples were located close to the testis samples based on the RPKM values of the mRNAs.

To examine the tissue-specific expression patterns of lncRNAs across distinct tissues, we first calculated the specificity indices of mRNAs and lncRNAs for nine tissue samples based on the definition of the Jensen-Shannon (JS) divergence score. Noticeably, the JS score for mRNA peaked at approximately 0.3 in the density plot, while the peak for lncRNA lagged behind that for mRNA (approximately 0.35). The median JS scores for mRNA and lncRNA were 0.385 and 0.422, respectively, suggesting that both mRNA and lncRNA exhibited a clear spatial-specific expression pattern across distinct tissues. Statistical analysis indicated that the specificity for lncRNA was significantly higher than that for mRNA (Fig. 3b, *P* < 2.2e-16, Kolmogorov-Smirnov test). In addition, we calculated the JS scores of lncRNAs and mRNAs for the samples at different developmental stages. Similarly, lncRNAs showed much higher specificity scores than mRNAs across the developmental periods (Fig. S6, *P* < 2.2e-16, Kolmogorov-Smirnov test). To avoid the bias of JS scores potentially caused by the expression level, we also compared the JS scores for lncRNAs and mRNAs with similar expression levels. PCGs and lncRNAs were divided into three groups based on the maximum RPKM value for each gene across nine samples (low: RPKM_max_ < 5.0; moderate: 5.0 ≤ RPKM_max_ < 50.0; high: RPKM_max_ ≥ 50.0). Subsequently, we calculated the specificity scores for coding genes and lncRNAs belonging to the three groups separately. Interestingly, only a minor difference was found between mRNAs and lncRNAs for the transcripts showing low expression, while a larger difference in the tissue specificity score between mRNAs and lncRNAs was observed for the moderately and highly expressed transcripts, especially for the transcripts with a moderate expression level (Fig. 3c). In addition, we computed the tau index for mRNAs and lncRNAs across nine samples. The results were almost the same as those of JS scores. On the whole, lncRNAs showed significantly stronger tissue specificity than mRNAs (Fig. S7A). For three groups with different expression levels, the similar trend was observed (Fig. S7B).

Furthermore, we defined the tissue possessing the maximum expression level as the tissue showing specific expression. We counted the number of specifically expressed lncRNAs and compared the distribution of the specificity scores across different tissues (Fig. S8). Strikingly, the testis, female antennae and accessory gland represented the top three tissues with the most specifically expressed lncRNAs. The accessory gland and testis were representative of tissues with the highest specificity scores. By contrast, the ovary exhibited the lowest specificity scores. Collectively, lncRNAs showed a more significant spatiotemporally specific expression pattern than mRNAs in the codling moth.

### Differential expression and sex-biased expression pattern of lncRNAs

**Fig. 4:**
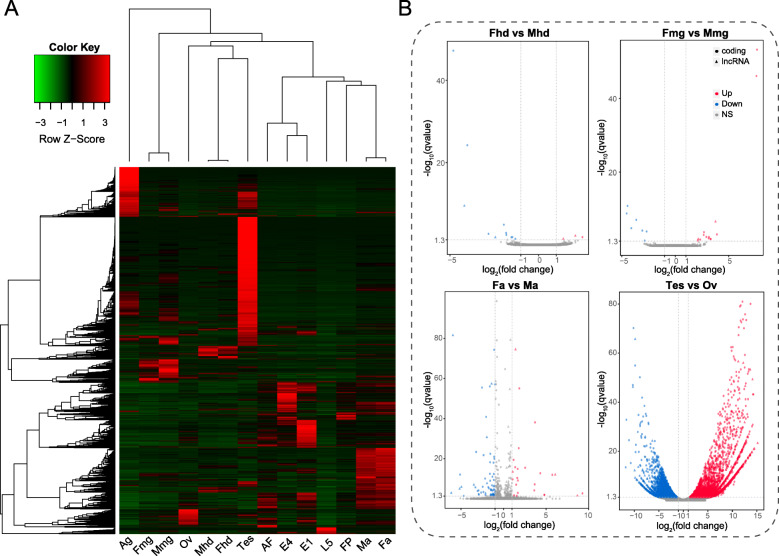
Differential expression analysis and identification of sex-biased lncRNAs. **a** Hierarchical clustering heat map showing the expression profile of *C. pomonella* lncRNAs across seven different tissues and developmental stages. Apparently, the expression profile of lncRNAs exhibits a tissue-specific pattern. Abbreviations are listed as follows: Ag, accessory gland; Ma, male antennae; Fa, female antennae; Tes, testis; Ov, ovary; Mhd, male head; Fhd, female head; Mmg, male midgut; Fmg, female midgut; E1, egg day 1; E4, egg day 4; L5, 5th-instar larva; FP, female pupa; AF, adult female. **b** Volcano plots showing differentially expressed genes in sex-matched tissue samples. As shown in the figure, there was no significant difference in expression between males and females in the head, midgut, and antenna samples, and only a few genes were found to be differentially expressed between the male and female samples. By contrast, a large number of genes were found to be differentially expressed between the testis and ovary, suggesting a large difference in expression between the testis and ovary. Remarkably, many more lncRNAs were upregulated in the testis than in the ovary

To explore the differentially expressed lncRNAs in the codling moth, we performed pairwise comparisons of RNA-seq samples from different tissues and various developmental stages. Differential gene expression analysis was conducted for each pairwise comparison of the tissue samples except for female and male antennae. For the RNA-seq data of samples from different developmental stages, differential expression analysis was performed only between adjacent stages. As illustrated in Fig. 4a, the differentially expressed lncRNAs could be clustered into several distinct groups based on their expression levels.

Next, we sought to investigate whether a sex-biased pattern existed in the codling moth. For the determination of sex-biased genes, we focused on the sex-matched samples, i.e., the tissue samples from both male and female insects. Figure 4b shows the volcano plots of the differentially expressed genes (DEGs) identified for each pair of sex-matched samples (female head vs. male head, female midgut vs. male midgut, female antennae vs. male antennae, and testis vs. ovary). After the application of the filtering criteria (|log2FC| > 1 and adjusted *p*-value < 0.05), few DEGs remained for most sex-matched samples, with the exception of the reproduction-related organs (testis vs. ovary) (bottom right). Thus, we placed more emphasis on the reproductive organ samples. No significant difference in number of PCGs with sex-biased expression was observed between the two sexes (male vs. female: 2654 vs. 2640). On the other hand, there were more male-biased lncRNAs than female-biased lncRNAs (male vs. female: 2287 vs. 455). Furthermore, we found that the male-biased tendency of lncRNAs was statistically significant compared to that of PCGs (*P* < 2.2e-16, Fisher’s exact test), suggesting that the lncRNAs specifically expressed in the testis might play a role in the process of spermatogenesis.

### Stronger homologous relationship based on synteny than sequence conservation for codling moth lncRNAs

To examine the sequence conservation of codling moth lncRNAs across insect species, BLASTN searches were first conducted against several insects with an E-value of 1e-3, including *A. aegypti*, *Anopheles gambiae*, *D. melanogaster*, *A. mellifera*, *Nilaparvata lugens*, *B. mori*, and *P. xylostella*, for the identification of the homologous counterparts of CplncRNAs in target insects. The results showed that a small fraction of CplncRNAs presented significant hits in target genomes. Specifically, significant hits in the abovementioned insects were obtained for 22, 15, 15, 10, 8, 461, and 812 CplncRNAs (Table 1), respectively. According to the taxonomic groups of these insects, 4.66 ~ 8.22% of the total CplncRNAs exhibited homologous counterparts in lepidopteran insects (461/9875 for *B. mori*, 812/9875 for *P. xylostella*), while only 0.08 ~ 0.22% of the CplncRNAs exhibited similar sequences in non-lepidopteran insects (8/9875 for *N. lugens*, 22/9875 for *A. aegypti*). Interestingly, the number of BLAST hits seemed to be positively correlated with the phylogenetic distance between the pairs of insect species.

**Table 1:** Sequence conservation analysis of CplncRNAs across insect species

Species	Order	#CplncRNAs with BLAST hits against target lncRNAs^**a**^	#homologous lncRNAs in target species^**b**^	#CplncRNAs with BLAST hits against target genome^**c**^	Reference
*Aedes aegypti*	Diptera	10/8	9/8	22/11	[14]
*Anopheles gambiae*	Diptera	0/0	0/0	15/5	[12]
*Drosophila melanogaster*	Diptera	1/1	1/1	15/11	[11]
*Apis mellifera*	Hymenoptera	1/1	1/1	10/6	[15]
*Nilaparvata lugens*	Hemiptera	0/0	0/0	8/0	[17]
*Bombyx mori*	Lepidoptera	74/58	488/349	461/236	[16]
*Plutella xylostella*	Lepidoptera	129/108	153/116	812/467	[18]

^a^ The number of CplncRNAs that exhibit BLAST hits against lncRNAs identified in target insect

^b^ The number of homologous lncRNAs that have significant hits with CplncRNAs in target insect

^c^ The number of CplncRNAs that show BLAST hits against target genome

^*^The numbers before the symbol ‘/’ represent the result for E-value cutoff of 1e-3, and the numbers after the symbol ‘/’ stand for the result for E-value cutoff of 1e-10

To determine whether these BLAST hits were located in regions encoding lncRNA loci, the CplncRNAs were used for direct BLASTN searches against lncRNA transcripts in target insects. For lepidopteran insects, 74 and 129 CplncRNAs were found to show significant hits to 488 and 153 lncRNAs in *B. mori* and *P. xylostella*, respectively. For non-lepidopteran insects, ten CplncRNAs exhibited significant hits to nine lncRNAs in *A. aegypti*, and one CplncRNA showed significant sequence similarity with one lncRNA in each of *D. melanogaster* and *A. mellifera*, while no CplncRNAs showed significant similarity with lncRNAs in either *A. gambiae* or *N. lugens*. These results indicated that a smaller percentage (0 ~ 16.05%) of the homologous counterparts of CplncRNAs overlap with lncRNA loci in target insects (0 for *A. gambiae* and *D. lugens*, 74/461 for *B. mori*).

Subsequently, we used a more stringent E-value cutoff (1e-10) to perform BLASTN search against those insect genomes. Clearly, the hit numbers in target insect genomes decreased by 26.67 ~ 100% (4/15 for *D. melanogaster*, 8/8 for *N. lugens*). Similarly, the number of homologous counterparts of CplncRNAs overlapping lncRNA loci in target species reduced by 16.28 ~ 21.62% (21/129 for *P. xylostella*, 16/74 for *B. mori*). Overall, fewer homologous counterparts of CplncRNAs in other insects were identified using a more strict E-value.

In addition to sequence similarity, syntenic relationships in the genomic context can reflect the conservation of lncRNAs between examined species. Herein, we specifically performed a comparative analysis between *C. pomonella* and *B. mori* and classified their lncRNA loci into families using a previously reported pipeline with some modifications. For the synteny analysis, six adjacent PCGs of a certain lncRNA (three on each side) were considered. LncRNAs from two species sharing at least three adjacent PCGs with orthologous relationship and at least one PCG on each side were grouped into a family. Strikingly, a total of 833 families were identified for lncRNA loci between these two species (Table S6), encompassing 1144/1240 and 1178/2531 lncRNA loci/transcripts in *C. pomonella* and *B. mori*, respectively. Compared with sequence similarity based on BLASTN searches, synteny analysis significantly increased the number of lncRNAs with homologous relationships between these two species (from 74 to 1240 in *C. pomonella*; from 488 to 2531 in *B. mori*). Taken together, the results indicated that lncRNAs showed weak sequence conservation across insect species, especially for insects with long evolutionary distances.

### Higher correlation of lncRNAs with neighbouring PCGs in *C. pomonella*

**Fig. 5:**
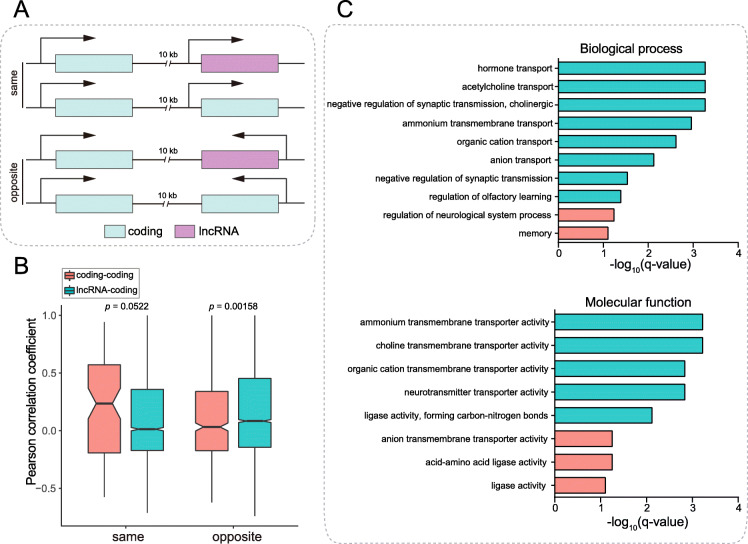
Correlation of the expression levels of lncRNAs with neighbouring protein-coding genes. **a** Schematic diagram of lncRNA/coding and neighbouring coding genes within a 10 kb distance. The upper panel shows the lncRNA-coding and coding-coding GPs with the same transcription direction (same-direction group), and the lower panel represents GPs with opposite transcription directions (opposite-direction group). **b** Box plot showing the distribution of the PCCs of coding-coding and lncRNA-coding GPs with the same or opposite transcription directions. **c** GO enrichment results for coding genes overlapping with neighbouring lncRNAs located on opposite strands for biological processes (top) and molecular functions (bottom). GO enrichment analysis was performed using the clusterProfiler package. Enriched GO terms with q-values < 0.05 were defined as statistically significant. Orange indicates significantly enriched GO terms, while blue indicates GO terms that were weakly or not enriched

To investigate the relationship of lncRNAs with their neighboring PCGs in expression, we searched the neighbouring PCGs within a 10-kb distance upstream or downstream of lncRNAs. For the determination of neighbouring coding genes, the PCGs overlapping with lncRNAs were also included for further analysis. The majority of the lncRNAs (6831, 69.17%) were found to exhibit neighbouring coding genes. A total of 3199 of the lncRNA-coding gene pairs (GPs) showed an absolute value of PCC > 0.5; thus, these lncRNAs represent potential candidates with *cis*-regulatory roles. As a control, the adjacent coding genes of the PCGs were also identified at the genome scale. Coding-coding GPs showed higher PCCs than lncRNA-coding GPs (Fig. S9A, *P* = 2.2e-16, Wilcoxon rank sum test). Subsequently, comparative analysis was separately conducted for non-overlapping and overlapping GPs. Among non-overlapping GPs, coding-coding GPs also showed remarkably higher PCCs than lncRNA-coding GPs (Fig. S9B, *P* < 2.2e-16, Wilcoxon rank sum test). Among overlapping GPs, however, no significant difference in the correlation level was observed between lncRNA-coding and coding-coding GPs (Fig. S9C, *P* = 0.1499, Wilcoxon rank sum test). Furthermore, the overlapping GPs were divided into two groups (one exhibiting the same transcription direction, referred to as the same-direction group, while the other showed the opposite transcription direction and was referred to as the opposite-direction group), as shown in Fig. 5a. The results revealed that the PCC of the lncRNA-coding GPs was significantly higher than that of the coding-coding GPs in the opposite-direction group (Fig. 5b, *P* = 0.00158, Wilcoxon rank sum test), while no difference was observed between two classes of GPs in the same-direction group (Fig. 5b, *P* = 0.0522, Wilcoxon rank sum test).

To explore the functions of PCGs in lncRNA-coding GPs, gene ontology (GO) enrichment analysis was performed for the highly correlated GPs (|PCC| > 0.5). The results showed that a variety of biological processes were closely associated with PCGs overlapping with lncRNAs, such as hormone transport, acetylcholine transport, the negative regulation of synaptic transmission, ammonium transmembrane transport, cation and anion transport, and the regulation of olfactory learning, while the regulation of neurological system process and memory were weakly related (Fig. 5c, top). Additionally, these PCGs were significantly implicated in a subset of molecular functions involving transporter activity for multiple substances, including ammonium, choline, organic cations and neurotransmitters, and ligase activity for forming carbon-nitrogen bonds (Fig. 5c, bottom). In contrast to the overlapping GPs, no significantly enriched GO terms were found for the PCGs within non-overlapping GPs.

Remarkably, more lncRNA-coding GPs showed positive correlations than negative correlations (|PCC| > 0.5, positive: 3108, negative: 91). Intriguingly, nineteen lncRNA-coding GPs showed a PCC < − 0.60 (Fig. S10 and S11), potentially suggesting that they exert negative impacts on the expression of the neighbouring coding genes. Nevertheless, whether these lncRNAs are authentic negative regulators of the gene expression of neighbouring PCGs remains to be experimentally investigated. Overall, a substantial proportion of lncRNAs were found to exhibit a stronger correlation with neighbouring PCGs and might play *cis*-acting roles in the regulation of gene expression.

## Discussion

In recent years, numerous studies have reported the identification and characterization of lncRNAs in insects. In this work, we identified and characterized an expanded landscape of lncRNAs in an invasive pest, the codling moth. A series of public RNA-seq datasets that are currently available for *C. pomonella*, encompassing multiple tissues and developmental stages, were employed for lncRNA discovery to achieve a higher accuracy and coverage. Subsequently, a detailed comparative analysis between lncRNAs and PCGs was conducted to reveal the characteristics of the lncRNAs from multiple aspects. Additionally, lncRNAs were characterized in terms of tissue specificity, sequence conservation across insect species, the correlation with neighbouring coding genes in terms of expression, and the coexpression network. This work advances our understanding of the composition and potential roles of lncRNAs in the codling moth.

In this study, a total of 9875 candidate lncRNA transcripts were identified in *C. pomonella* (Table S2). Nevertheless, the catalogue of lncRNAs identified in this study was limited. First, the datasets used in this study did not include samples from all tissues or developmental stages or any specific treatment. It can be anticipated that more lncRNAs will be identified when more RNA-seq datasets are available under different conditions and treatments. Second, the RNA-seq data used in this study relied on the use of magnetic beads for enrichment of polyadenylated transcripts; thus, non-polyadenylated transcripts, which might account for a large proportion of lncRNAs, were lacking. The majority of non-polyadenylated lncRNAs would be retained by employing an rRNA-depletion plus strand-specific RNA-seq strategy. Additionally, monoexonic transcripts, which were absent in our analysis, could be confidently identified based on strand-specific sequencing. Third, with the development of computational approaches and bioinformatics pipelines for distinguishing noncoding from coding RNAs [29, 30], particularly convolutional neural network-based deep learning algorithms [31], noncoding RNAs could be identified more accurately. Fourth, most of the pipelines for lncRNA identification rely on transcriptome reconstruction from millions of Illumina short reads, resulting in a large proportion of incomplete or fragmented transcripts. It can be envisioned that the completeness of lncRNAs will be remarkably improved with the application of PacBio SMRT or Nanopore sequencing strategies [32, 33]. Additionally, the numbers of lncRNAs reported in various insects varied greatly, this might be caused by multiple factors, such as sequencing depth, different analysis approaches, and parameter settings.

In this study, the characteristics of lncRNAs were revealed through comparison with mRNAs. It was shown that the flanking sequences of splice sites were highly similar between mRNA and lncRNA, suggesting that they might share a common splicing mechanism. In addition, lncRNAs were significantly shorter than mRNAs in transcript length, which is in accordance with previous findings. Further analysis suggested that exon number had a major effect, while exon size showed a minor but statistically significant effect on the difference in transcript size between mRNA and lncRNA. With regard to the expression level, our data supported the notion that lncRNAs are expressed at low levels compared to mRNAs, while antisense lncRNAs tend to be more actively transcribed. Interestingly, our data indicated that lncRNAs were more likely to be associated with TEs than PCGs, supporting the notion that most lncRNAs might originate from TEs in genome.

It is generally accepted that the expression of many genes exhibits tissue specificity, which usually indicates the distinct roles of genes functioning in specific tissues or organs. Our results suggested that lncRNAs were more likely to exhibit tissue-specific expression than PCGs (Fig. 3b). Additionally, lncRNAs appeared to present more highly tissue-specific expression than PCGs in moderate and high expression groups (Fig. 3c). We considered this pattern to be reasonable, partly because PCGs with moderate and high expression are commonly housekeeping genes or necessary for maintaining basic physiological activity in organisms. Although the JS score at the peak position was significantly higher in lncRNAs than in mRNAs, we observed a small peak for mRNAs in the right tail of the density plot (Fig. 3b). The small peak probably represented a set of tissue-specific PCGs. More interestingly, these highly tissue-specific PCGs were mainly derived from genes with low expression, because a similar peak was found for mRNAs in the low expression groups, while no such peak was found in moderate and high expression groups (Fig. 3c). Furthermore, we examined how many highly tissue-specific PCGs were identifiable and the tissues showing the highest expression of these genes. According to the results, 493 PCGs were identified as highly tissue-specific genes, among which the majority (478, 96.96%) belonged to the low expression group, and 14 (2.84%) belonged to the moderate expression group, while only one (0.2%) belonged to the high expression group, in agreement with the results presented in Fig. 3c. In addition, most of these highly tissue-specific PCGs were found to be expressed in a single tissue, while no detectable expression was found in other tissues. The statistical results indicated that the antennae (192, 38.95%) and male reproductive organs (testis and accessory gland, 132, 26.77%) were the main tissues in which the expression of these highly tissue-specific PCGs was observed.

In addition to the tissue-specific pattern across all tissues, we compared gene expression between sex-matched tissue samples. Minor differences were observed for paired nonreproductive tissues, such as the head, midgut, and antennae, suggesting no significant sex bias in gene expression for these tissues (Fig. 4b). However, a large number of PCGs and lncRNAs showed significant variations in expression levels between the testis and ovary. For PCGs, we found that the number of upregulated genes was comparable to that of downregulated genes in the testis relative to the ovary. By contrast, more lncRNAs appeared to show biased expression in the testis than in the ovary, possibly implying the important roles of lncRNAs in spermatogenesis. Recently, the critical roles of lncRNAs in the male reproductive system of *Drosophila* have been investigated. Maeda et al. reported a male-specific abdominal lncRNA that plays a key role in *Drosophila* accessory gland development and male fertility [19]. Additionally, Wen et al. reported the identification of 128 testis-specific lncRNAs in *Drosophila* and demonstrated that knocking out these lncRNAs could cause a significant loss of male fertility and abnormality of sperm development [20]. Our findings could serve as a supplement to previous research indicating the prevalence of testis-specific lncRNAs. However, the mechanistic roles of testis-specific lncRNAs in the codling moth require further investigation through gain-of-function or loss-of-function studies.

BLASTN-based sequence similarity analysis demonstrated that codling moth lncRNAs exhibit low sequence conservation across insect species. This poor sequence conservation means that most lncRNAs are unique in different species, which is consistent with the findings of a previous study in which most of the conserved lncRNAs only occurred between two pairs of species in the same genus [34]. Thus, they concluded that whether conserved lncRNAs existed was heavily dependent on the divergence time between species [34]. Regarding the low conservation of lncRNAs, multiple hypotheses have been raised in previous studies [35–38]. First, the most straightforward hypothesis might be related to the rapid evolution of lncRNAs [37]. It has been postulated that there is less selective pressure on lncRNAs due to their noncoding nature, resulting in great variation in the sequence of lncRNAs across organisms. Second, diversity in sequence does not necessarily guarantee functional divergence, for example, because functions may be maintained by the conserved secondary structure of lncRNAs [35, 37, 38]. Third, the origin of lncRNAs in the insertion of TEs might also contribute to the rapid changes in lncRNA sequences [36]. These hypotheses might partially explain the low sequence conservation of lncRNAs among insect species.

Alternatively, it has been reported that cross-species orthologous lncRNAs might be identified through synteny analysis on a genome-wide scale [39, 40]. Accordingly, we adopted a previously reported pipeline with some modifications for the identification of syntenic lncRNAs between *C. pomonella* and *B. mori* [41]. The core idea was to judge the homologous relationship of interspecific lncRNAs based on shared neighbouring PCGs with orthologous relationships. Interestingly, the ratio of lncRNAs with homologous relationships between these two species was greatly increased in comparison to the results of the sequence similarity-based method. The results suggested that synteny could be effectively used as a proxy for the conservation of lncRNAs between different species. Therefore, synteny might be considered a more reliable standard for determining the homologous relationship of lncRNAs among insect species.

Due to the lack of protein-coding capacity, it remains a great challenge to predict the functional roles of lncRNAs in physiological processes. Previously, the ‘guilt-by-association’ principle has been widely accepted for the inference of lncRNA function [42–45]. An increasing number of studies support the notion that lncRNAs are likely to regulate the expression of neighbouring PCGs within a 10 kb distance, referred to as *cis-*acting functions. In this study, we compared the correlation between neighbouring lncRNA-coding and coding-coding GPs. For all neighbouring GPs, coding-coding GPs showed a more significant correlation than lncRNA-coding GPs. In our opinion, this finding is reasonable. In most cases, PCGs actually act as the direct executors of functions in cells, resulting in the good coordination of gene expression. Similar results were observed for non-overlapping GPs. Interestingly, for overlapping GPs, lncRNA-coding GPs exhibited a higher correlation than coding-coding GPs in the opposite-direction group, while no significant difference was observed between lncRNA-coding and coding-coding GPs in the same-direction group. Thus, lncRNA-coding GPs in the opposite-direction group are likely to represent *cis*-acting GPs. GO enrichment analysis showed that opposite coding genes overlapping with lncRNAs were strongly associated with many biological processes, such as hormone transport, acetylcholine transport and the regulation of olfactory learning, implying that lncRNAs function in a *cis*-regulatory manner. In addition, we also wondered why more lncRNA-coding GPs showed a positive correlation than a negative correlation. Theoretically, lncRNAs, particularly those transcribed on the complementary strand, might interfere with the expression of neighbouring PCGs. One possible explanation might be that the gene expression of PCGs is probably also influenced by long-range interactions of *trans*-acting lncRNAs. Collectively, our findings highlighted a set of lncRNAs as promising regulators of gene expression in a *cis*-acting manner.

## Conclusions

In summary, a comprehensive set of lncRNAs has been characterized in the codling moth on the genomic scale. The generation of more publicly available RNA-seq datasets would contribute to the discovery of a broad variety of novel lncRNAs in this insect. The genomic features of codling moth lncRNAs are demonstrated to be distinct from those of PCGs based on detailed pairwise comparisons. Additionally, expression pattern analysis shows the higher tissue specificity of lncRNAs relative to coding genes. The poor sequence conservation of lncRNAs across insect species reflects either the rapid evolution of lncRNAs or the low dependence of their functional roles on their primary sequences. Importantly, synteny appears to be a better standard for the identification of lncRNAs with homologous relationships between species. Furthermore, the correlation of lncRNAs with their neighbouring PCGs highlights the potential impacts of lncRNAs on the regulation of gene expression. Overall, our work provides a valuable resource for the comparative and functional study of lncRNAs in the codling moth, which will help to elucidate their mechanistic roles in transcriptional regulation.

## Methods

### Collection of RNA-seq datasets

In this study, three publicly available RNA-seq datasets for *C. pomonella* were downloaded from the NCBI SRA (Sequence Read Archive) database (Table S1), including 14 samples from seven tissues including two biological replicates for each (female head, male head, female midgut, male midgut, testis, ovary, accessory gland) from study SRP083782 (SRR4101328-SRR4101341) [46], two samples without biological replicates (male and female antennae) from study SRP060413 (SRX1082030, SRX1082029) [47], and five samples from different developmental stages (two embryonic stages, one larval stage, pupa and adult stage) from our previous study SRP181710 (SRR8479435, SRR8479438, SRR8479439, SRR8479440, SRR8479441) [28].

### Mapping to the reference genome and transcriptome assembly

The quality of the Illumina RNA-seq datasets was checked using FastQC (http://www.bioinformatics.babraham.ac.uk/projects/fastqc/). Raw reads were trimmed to obtain clean data by removing adaptors and low-quality or short reads using Trimmomatic v1.3 [48]. For each sample, paired-end clean reads were separately mapped onto the genome using GSNAP version 2019-06-10 [49] with the default parameters. SAMtools v1.5 [50] was used to count the number of mapped reads. Then, StringTie v1.3.3b [51] was employed to assemble transcripts for each sample. Subsequently, multiple transcript datasets from all samples were combined into a single dataset using the merge function within StringTie, yielding a merged dataset containing the transcripts found in all samples. The assembled transcripts were compared with the reference annotation for their classification using gffcompare v0.10.1.

### Bioinformatics analysis pipeline for the identification of lncRNAs

To annotate lncRNAs in the genome of *C. pomonella*, we employed an analysis pipeline developed by our group. After transcriptome assembly, a large proportion of the transcripts fully overlapped (‘=’) or partially overlapped (‘j’) with known PCGs and were thus excluded from the subsequent analysis. The newly assembled transcripts were selected for the identification of lncRNAs, including those completely located in intergenic regions (“u”), those showing exonic overlap with PCGs on the opposite strand (“x”), and those fully contained within a reference intron (“i”). Subsequently, the transcripts that were putatively considered to be lncRNAs had to meet the following stringent criteria: a) the transcript is ≥200 nt in size and has at least two exons; b) the transcript shows no or weak protein-coding potential based on the prediction results of CPC (Coding Potential Calculator) [52] and CNCI (Coding Non-Coding Index) [29], which are two commonly used software programs for the evaluation of the coding potential of transcripts; c) the predicted ORF size of a transcript is less than 300 nt; d) the transcript exhibits no significant hit (E-value = 1e-3) according to BLASTX search against the SwissProt protein database; e) no significant hits (E-value = 1e-3) were found for the transcript based on a HMMER3 search against the Pfam protein domain database (release Pfam31.0) [53]; and f) transcripts with low abundance (RPKM_max_ < 1.0) are discarded. Based on the class codes assigned by gffcompare, the lncRNAs were divided into three categories: lincRNAs (class code “u”), ilncRNAs (class code “i”), and lncNATs (class code “x”).

### Tissue-specific expression analysis of transcripts

To measure the level of tissue specificity for each gene, we calculated the JS divergence score [40] as a specificity index across nine different tissues or five developmental stages. For each group, we computed the average value from all replicates and calculated the JS score. Generally, the JS score ranges between 0 and 1, and larger values indicate more highly specific genes. To avoid the bias caused by the expression levels, coding genes and lncRNAs were categorized into three groups based on the maximum RPKM value for each gene across nine samples (low: RPKM_max_ < 5.0; moderate: 5.0 ≤ RPKM_max_ < 50.0; RPKM_max_ ≥ 50.0), and we computed JS scores for three groups, respectively. A previous benchmark study has shown that the tau index was a good metric for measuring tissue-specificity of gene expression [54], we also calculated tau index for coding genes and lncRNAs.

### Estimation of transcript abundance and differential expression analysis

To estimate the expression levels of PCGs and lncRNAs, HTSeq v0.10.0 [55] was employed to calculate the read counts that were mapped to the PCGs and lncRNA transcripts for each sample. To identify the genes that were differentially expressed in different tissues and developmental stages, differential expression analysis was conducted for each pair of samples with biological replicates using the R package DESeq2 [56]. For those samples without biological replicates (male and female antenna samples and samples collected at several developmental stages), the DEGseq package [57] was employed to perform the differential expression analysis, and TMM (trimmed mean of M-values) normalization was performed using the edgeR package [58] prior to the differential expression analysis. Raw read counts were used as the input data for both DESeq2 and DEGseq during the analysis. PCGs and lncRNAs with an adjusted *p*-value < 0.05 and a |log2fold-change| ≥ 1.0 were considered differentially expressed.

### Sequence conservation analysis of codling moth lncRNAs

To determine the sequence conservation level of codling moth lncRNA transcripts among insect species, we performed a comparative analysis of lncRNA sequences with several other insect species. The genome assemblies and gene annotations of seven representative insect species were collected for conservation analysis. The genome data of *A. aegypti* and *A. gambiae* were downloaded from VectorBase [59]. The genome sequence of *D. melanogaster* was downloaded from the UCSC Genome Browser website. The genome information of *B. mori* and *N. lugens* was obtained from InsectBase [60]. The genome of *P. xylostella* was downloaded from the diamondback moth genome database [61]. The genome sequence of *A. mellifera* was retrieved from BeeBase. The codling moth lncRNAs were searched against the genome of each insect using the BLASTN program with two E-value cutoffs (1e-3, and a more stringent threshold 1e-10).

### Synteny analysis of codling moth lncRNAs

To explore the syntenic relationship of interspecific lncRNAs, we classified the codling moth lncRNAs into families by adopting a Python pipeline (https://github.com/Gabaldonlab/Synthenic-Families) with some modifications as described previously [41]. Considering the similar method and comparable lncRNA number reported in *B. mori*, we attempted to identify the syntenic lncRNAs between the codling moth and the silkworm. First, we created a file including the orthologous relationships of all PCGs annotated in these two species using OrthoMCL software v2.0.9 [62] and an in-house Python script. Second, we generated a file including the pairwise syntenic relationships between lncRNAs from these two species using the modified synteny_nematodesv4GH.py script [41]. To fulfil the requirement of synteny, three adjacent genes on each side of a given lncRNA were considered. At least three shared genes and at least one shared gene on each side of a certain lncRNA were considered to be necessary conditions. Finally, lncRNAs from the two species were grouped into families using the modified classifyFamiliesv5_VennGH.py script [41].

### Correlation analysis of lncRNAs and neighbouring PCGs

To test the correlation of expression levels between lncRNAs and their closest PCGs, we identified the upstream and downstream coding genes of the lncRNAs within 10 kb in distance with the methodology described previously [35]. PCC was calculated using RPKM values with the *corr.test* function within the psych package in the R statistical environment.

### Functional enrichment analysis

To determine the functional relevance of coding genes that are potentially regulated by lncRNAs, we conducted functional enrichment analysis for the neighbouring coding genes of lncRNAs. For GO enrichment analysis, the gene list was taken as the query gene set for functional enrichment analysis using the enricher function for hypergeometric test within the clusterProfiler R package [63]. During the enrichment analysis, we provided the GO annotations ourselves. Subsequently, the *p*-values were corrected using the Benjamini-Hochberg (BH) method. Enriched GO terms with q-values < 0.05 were considered statistically significant.

### Statistical analysis

Unless otherwise stated, a two-tailed Wilcoxon rank sum test was conducted for the determination of statistical significance for the comparison of genomic features between PCGs and lncRNAs. Chi-squared test in R statistical environment was conducted for comparison of the distribution pattern of three classes of lncRNAs across three Lepidoptera insects. For the comparison of JS specificity scores, the Kolmogorov-Smirnov test was carried out to determine whether there was a significant difference between the two groups. Fisher’s exact test was performed when comparing the number of coding genes and lncRNAs that overlapped with TEs and analysing the number of transcripts with a male-biased expression pattern.

## Supplementary Information

**Additional file 1: Table S1.** Summary of RNA-seq datasets used in this study.

**Additional file 2: Table S2.** Genomic annotation of lncRNAs identified in the codling moth.

**Additional file 3: Table S3.** Genomic annotation of lncRNAs identified in *B. mori* using our pipeline.

**Additional file 4: Table S4.** Genomic annotation of lncRNAs identified in *P. xylostella* using our pipeline.

**Additional file 5: Table S5.** Distribution of coding moth lncRNAs on chromosomes.

**Additional file 6: Table S6.** LncRNA families between *C. pomonella* and *B. mori* according to synteny analysis.

**Additional file 7: Figure S1.** Bioinformatics pipeline for the identification of lncRNAs. The flowchart of lncRNA identification could be briefly summarized as: 1) clean reads mapping to the reference genome using GSNAP, 2) assembly of alignments into transcripts using StringTie for each sample, 3) merging of transcript sets from all samples into a single consensus transcript dataset, 4) eliminating transcripts overlapping or partially overlapping known transcripts on the sense strand to generate novel transcripts, 5) filtering out transcripts that are short (< 200 nt), monoexonic, and those with coding potential based on multiple strategies to obtain the putative lncRNA transcripts.

**Additional file 8: Figure S2.** Comparison of the distribution pattern of three classes of lncRNAs across three Lepidoptera insects. **(A)** The distribution pattern of three classes of codling moth lncRNAs was compared with those of *B. mori and P. xylostella* lncRNAs reported in the literature. Chi-squared test was used for determination of statistical significance. Due to the different classification types, non-lincRNAs and non-ilncRNAs in *C. pomonella* and *B. mori* were treated as the other type during Chi-squared test. **(B)** Comparison of the distribution pattern of lncRNAs identified in three Lepidoptera insects using our pipeline. Statistical significance was analyzed using Chi-squared test.

**Additional file 9: Figure S3.** Genomic distribution of lncRNAs across chromosomes in *C. pomonella*. **(A)** Circos plot showing the distribution of lncRNAs across 29 chromosomes, each panel represents a kind of genomic feature: (I) percentage of repetitive sequences in non-overlapping 200-kb windows; (II) density of PCGs in non-overlapping 200-kb windows; (III) density of lincRNAs in non-overlapping 200-kb windows; (IV) density of lncNATs in non-overlapping 200-kb windows; (V) density of ilncRNAs in non-overlapping 200-kb windows. **(B)** Correlation analysis of the number of lncRNAs on each chromosome and the corresponding chromosome size. Correlation analysis demonstrated that the number of lncRNAs was positively correlated with the chromosome size. **(C)** Correlation analysis of the number of lncRNAs on each chromosome and the number of PCGs on the corresponding chromosome. The number of lncRNAs was proportional to that of PCGs on the same chromosome.

**Additional file 10: Figure S4.** Sequence logos of the nucleotides flanking acceptor and donor sites of mRNAs (top) and lncRNAs (bottom) in the codling moth. Sequence logo was generated using the WebLogo software.

**Additional file 11: Figure S5.** Comparative analysis of exon number, exon size, intron size, and GC content between mRNAs and lncRNAs. **(A)** Frequency plot for the comparison of exon numbers between mRNAs and lncRNAs. **(B)** Box plot showing the distribution of exon sizes for mRNAs and lncRNAs. **(C)** Box plot presentation of the range of intron sizes for mRNAs and lncRNAs. **(D)** Frequency plot for the comparison of exon numbers between mRNAs and lncRNAs. The two-tailed Wilcoxon rank sum test was used for the determination of statistical significance.

**Additional file 12: Figure S6.** Density plot showing the distribution of tissue specificity scores for PCGs and lncRNAs in *C. pomonella* across different developmental stages.

**Additional file 13: Figure S7.** Density plot showing the distribution of tau index for PCGs and lncRNAs across nine tissue samples. **(A)** The distribution of tau index for all expressed PCGs and lncRNAs. **(B)** The distribution of tau index for PCGs and lncRNAs in three groups with different expression levels. Statistical significance of the difference in tau index between PCGs and lncRNAs was determined using the Kolmogorov-Smirnov test.

**Additional file 14: Figure S8.** Distribution pattern of the JS specificity scores of tissue-specific genes in different tissues.

**Additional file 15: Figure S9.** Distribution of PCCs for neighboring GPs. **(A)** Boxplot presentation of the distribution of PCCs for the entire set of neighboring gene pairs. **(B)** Boxplot showing the distribution of PCCs for non-overlapping gene pairs. **(C)** Boxplot representing the distribution of PCCs for overlapping gene pairs.

**Additional file 16: Figure S10.** Ten representative overlapping lncRNA-coding GPs showing negative correlation with PCC < − 0.6. Blue circle represents lncRNA, and orange circle indicates PCG. Abbreviations are listed as follows: Mhd, male head; Fhd, female head; Mmg, male midgut; Fmg, female midgut; Ma, male antennae; Fa, female antennae; Tes, testis; Ov, ovary; Ag, accessory gland; E1, egg day 1; E4, egg day 4; L5, 5th-instar larva; FP, female pupa; AF, adult female.

**Additional file 17: Figure S11.** Nine representative non-overlapping lncRNA-coding GPs showing negative correlation with PCC < − 0.6. Blue diamond indicates lncRNA, and orange square represents PCG. Abbreviations are listed as follows: Mhd, male head; Fhd, female head; Mmg, male midgut; Fmg, female midgut; Ma, male antennae; Fa, female antennae; Tes, testis; Ov, ovary; Ag, accessory gland; E1, egg day 1; E4, egg day 4; L5, 5th-instar larva; FP, female pupa; AF, adult female.

## Data Availability

The RNA-seq data used in this study could be accessed in the NCBI SRA database under the following accessions: SRP083782, SRP060413, and SRP181710.

## Abbreviations

LncRNA: Long noncoding RNA
PCC: Pearson correlation coefficient
lincRNA: Long intergenic noncoding RNA
ilncRNA: Intronic long noncoding RNA
lncNAT: Long noncoding natural antisense transcript
RPKM: Reads Per Kilobase of exon model per Million mapped reads
TE: Transposable element
CPC: Coding Potential Calculator
CNCI: Coding Non-Coding Index
GO: Gene ontology
PCG: Protein-coding gene
DEG: Differentially expressed genes
