# Supplementary figures and images for: The landscape of lncRNAs in Cydia pomonella provides insights into their signatures and potential roles in transcriptional regulation
Source: BMC Genomics. 2021 Jan 5;22:4. doi: 10.1186/s12864-020-07313-3 (PMC7786964; doi:10.1186/s12864-020-07313-3)

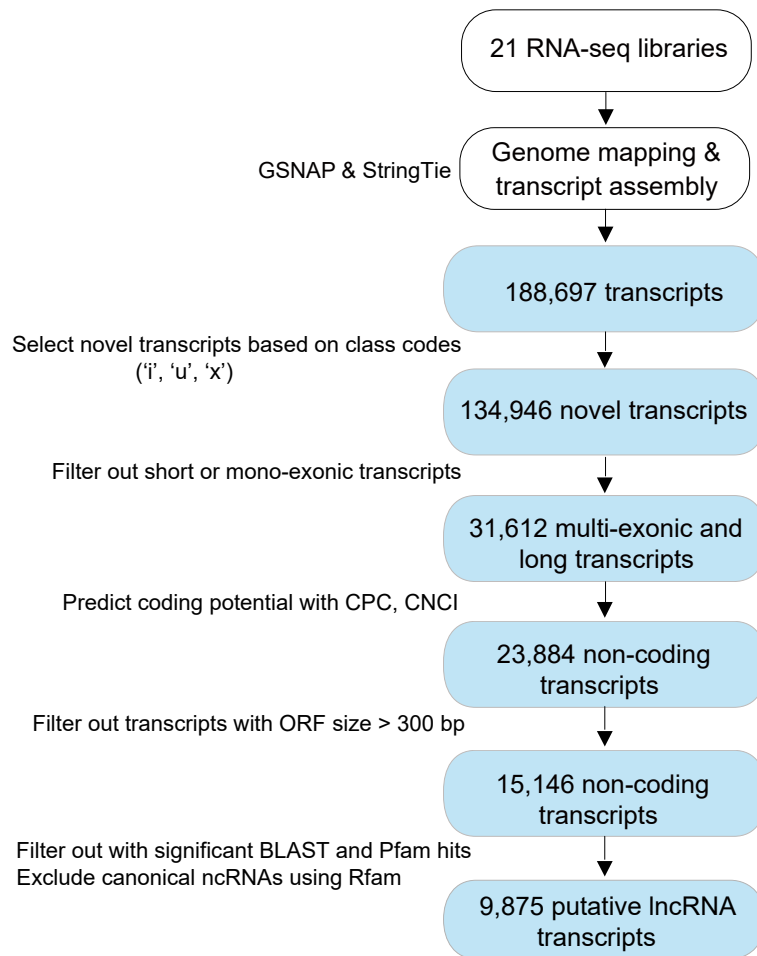

Supplement: Supplementary file 7 — Additional file 7: Figure S1. Bioinformatics pipeline for the identification of lncRNAs. The flowchart of lncRNA identification could be briefly summarized as: 1) clean reads mapping to the reference genome using GSNAP, 2) assembly of alignments into transcripts using StringTie for each sample, 3) merging of transcript sets from all samples into a single consensus transcript dataset, 4) eliminating transcripts overlapping or partially overlapping known transcripts on the sense strand to generate novel transcripts, 5) filtering out transcripts that are short (< 200 nt), monoexonic, and those with coding potential based on multiple strategies to obtain the putative lncRNA transcripts. [file 12864_2020_7313_MOESM7_ESM.pdf]

**A**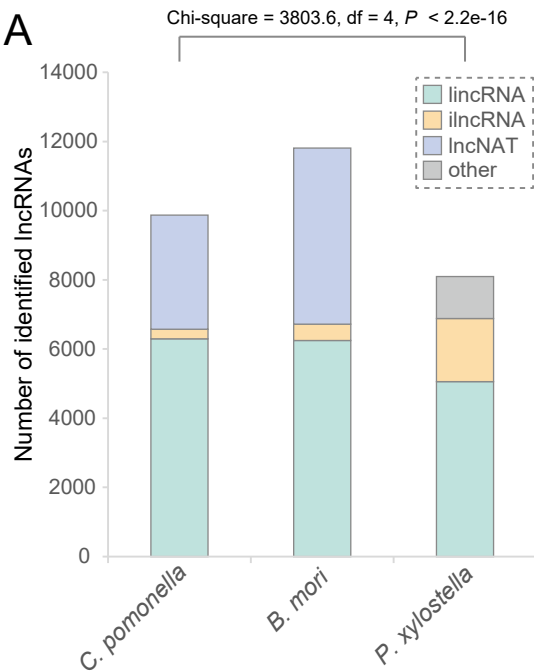**B**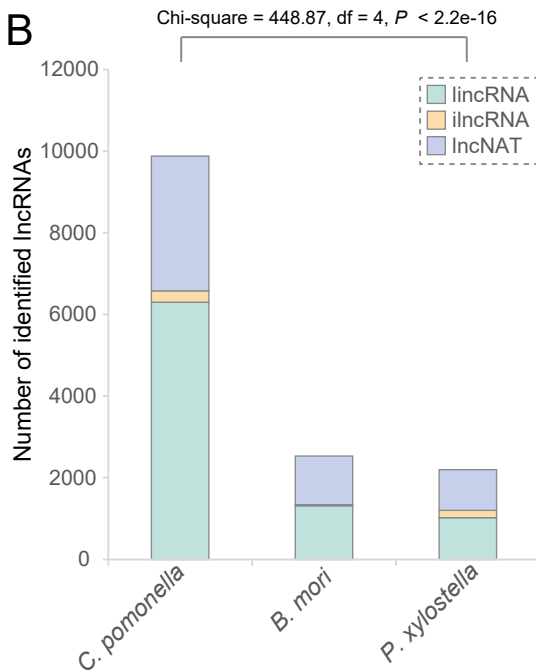

Supplement: Supplementary file 8 — Additional file 8: Figure S2. Comparison of the distribution pattern of three classes of lncRNAs across three Lepidoptera insects. (A) The distribution pattern of three classes of codling moth lncRNAs was compared with those of B. mori and P. xylostella lncRNAs reported in the literature. Chi-squared test was used for determination of statistical significance. Due to the different classification types, non-lincRNAs and non-ilncRNAs in C. pomonella and B. mori were treated as the other type during Chi-squared test. (B) Comparison of the distribution pattern of lncRNAs identified in three Lepidoptera insects using our pipeline. Statistical significance was analyzed using Chi-squared test. [file 12864_2020_7313_MOESM8_ESM.pdf]

A

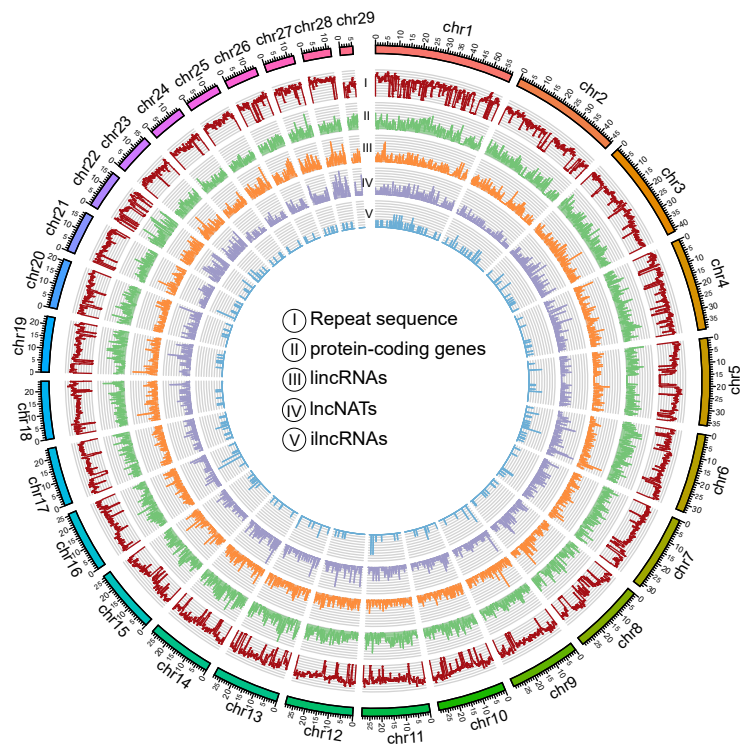

B

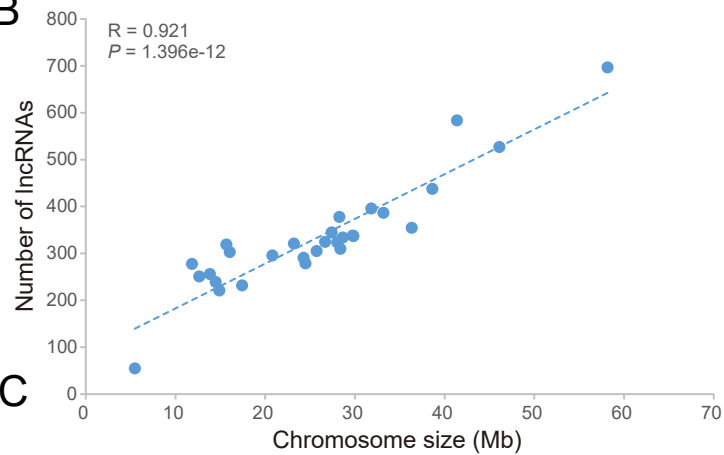

C

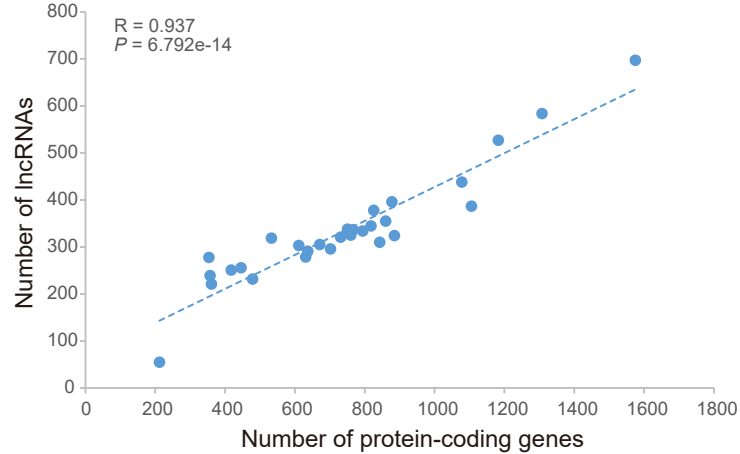

Supplement: Supplementary file 9 — Additional file 9: Figure S3. Genomic distribution of lncRNAs across chromosomes in C. pomonella. (A) Circos plot showing the distribution of lncRNAs across 29 chromosomes, each panel represents a kind of genomic feature: (I) percentage of repetitive sequences in non-overlapping 200-kb windows; (II) density of PCGs in non-overlapping 200-kb windows; (III) density of lincRNAs in non-overlapping 200-kb windows; (IV) density of lncNATs in non-overlapping 200-kb windows; (V) density of ilncRNAs in non-overlapping 200-kb windows. (B) Correlation analysis of the number of lncRNAs on each chromosome and the corresponding chromosome size. Correlation analysis demonstrated that the number of lncRNAs was positively correlated with the chromosome size. (C) Correlation analysis of the number of lncRNAs on each chromosome and the number of PCGs on the corresponding chromosome. The number of lncRNAs was proportional to that of PCGs on the same chromosome. [file 12864_2020_7313_MOESM9_ESM.pdf]

### Acceptor sites

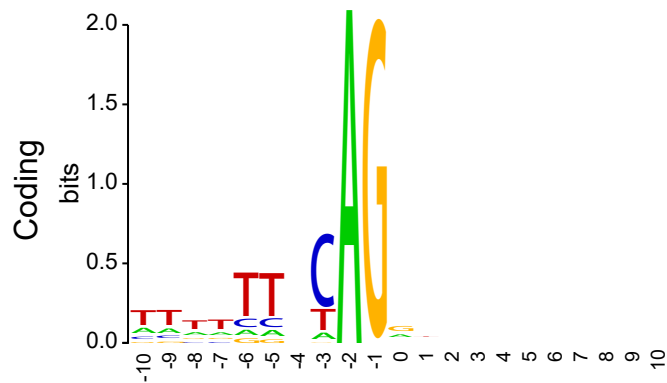

### Donor sites

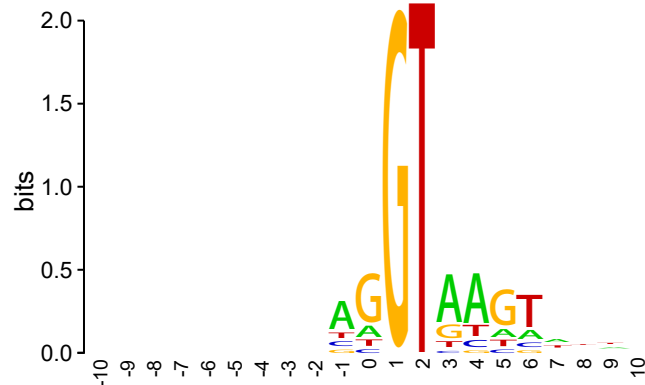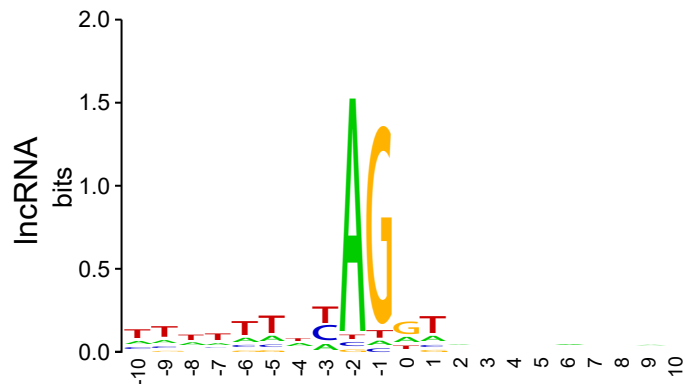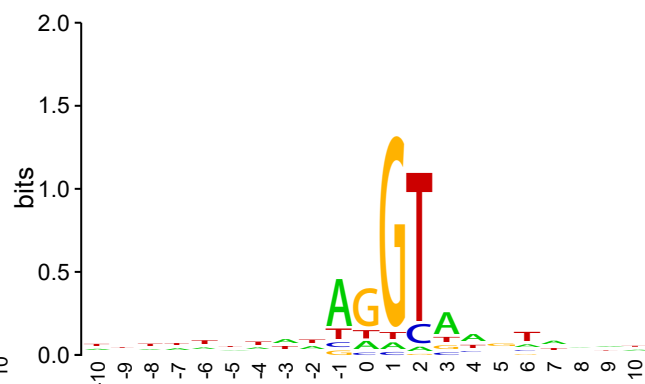

Supplement: Supplementary file 10 — Additional file 10: Figure S4. Sequence logos of the nucleotides flanking acceptor and donor sites of mRNAs (top) and lncRNAs (bottom) in the codling moth. Sequence logo was generated using the WebLogo software. [file 12864_2020_7313_MOESM10_ESM.pdf]

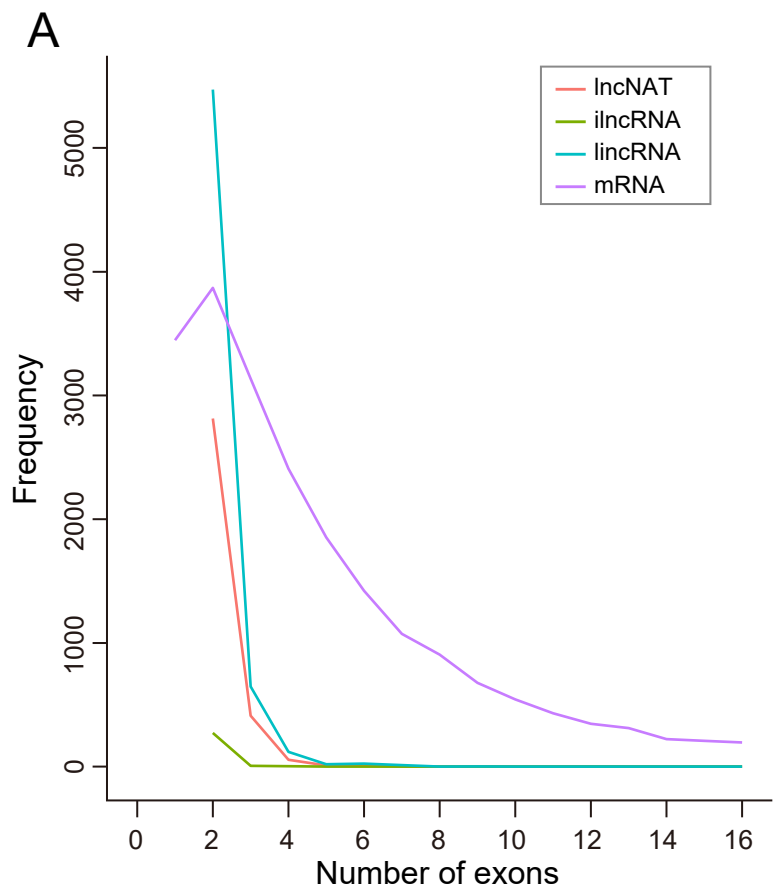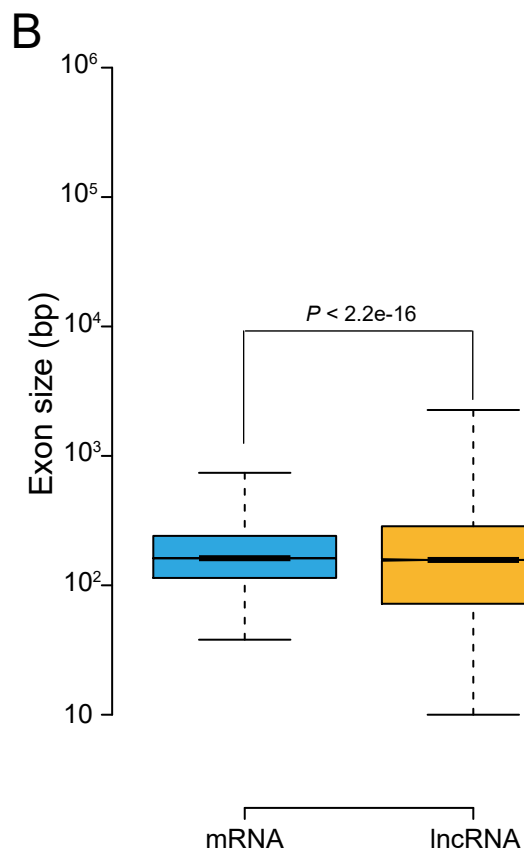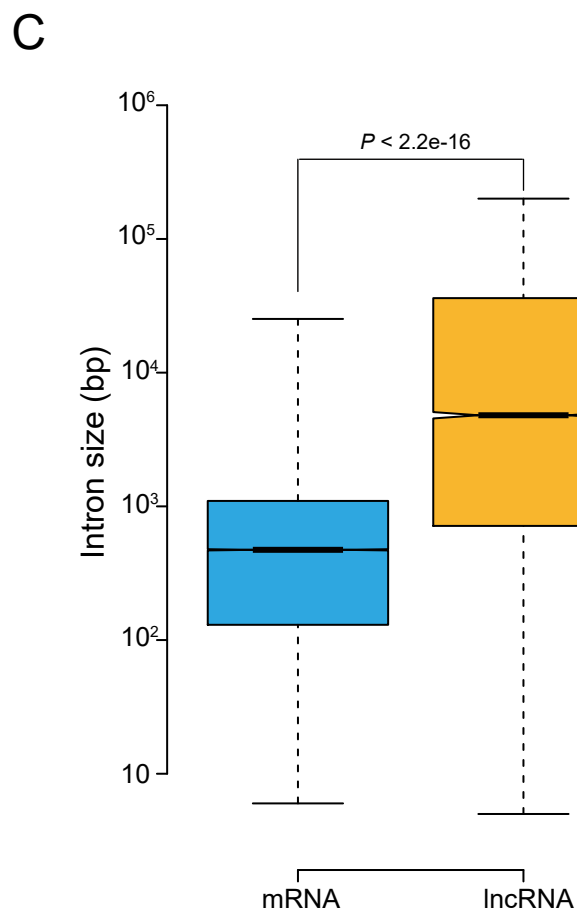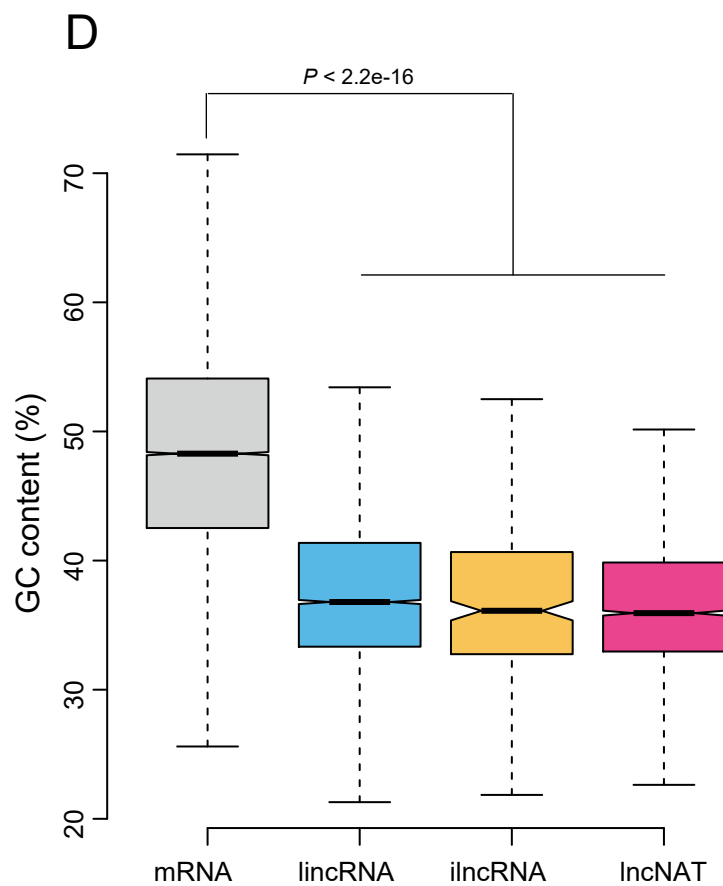

Supplement: Supplementary file 11 — Additional file 11: Figure S5. Comparative analysis of exon number, exon size, intron size, and GC content between mRNAs and lncRNAs. (A) Frequency plot for the comparison of exon numbers between mRNAs and lncRNAs. (B) Box plot showing the distribution of exon sizes for mRNAs and lncRNAs. (C) Box plot presentation of the range of intron sizes for mRNAs and lncRNAs. (D) Frequency plot for the comparison of exon numbers between mRNAs and lncRNAs. The two-tailed Wilcoxon rank sum test was used for the determination of statistical significance. [file 12864_2020_7313_MOESM11_ESM.pdf]

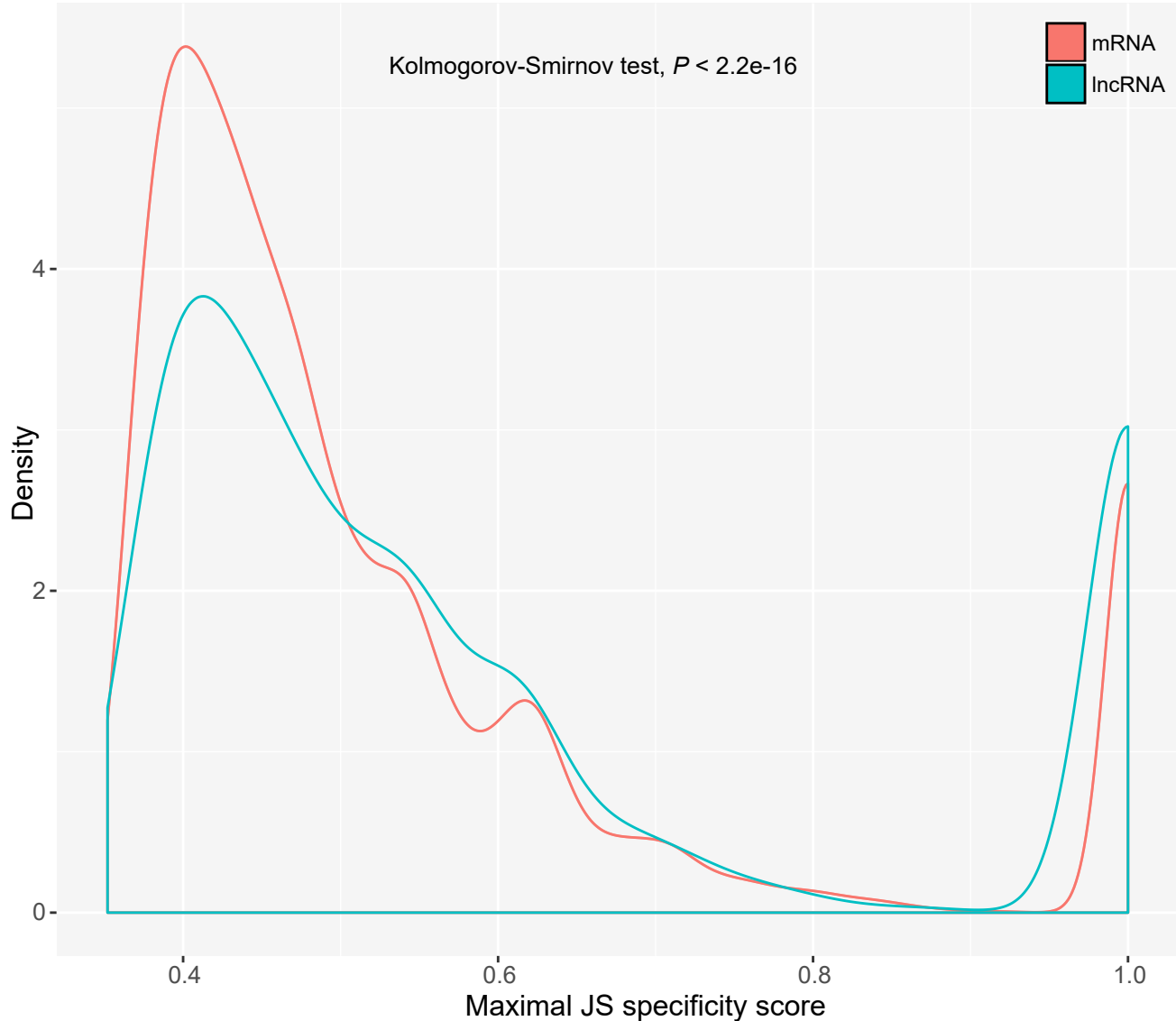

Supplement: Supplementary file 12 — Additional file 12: Figure S6. Density plot showing the distribution of tissue specificity scores for PCGs and lncRNAs in C. pomonella across different developmental stages. [file 12864_2020_7313_MOESM12_ESM.pdf]

A

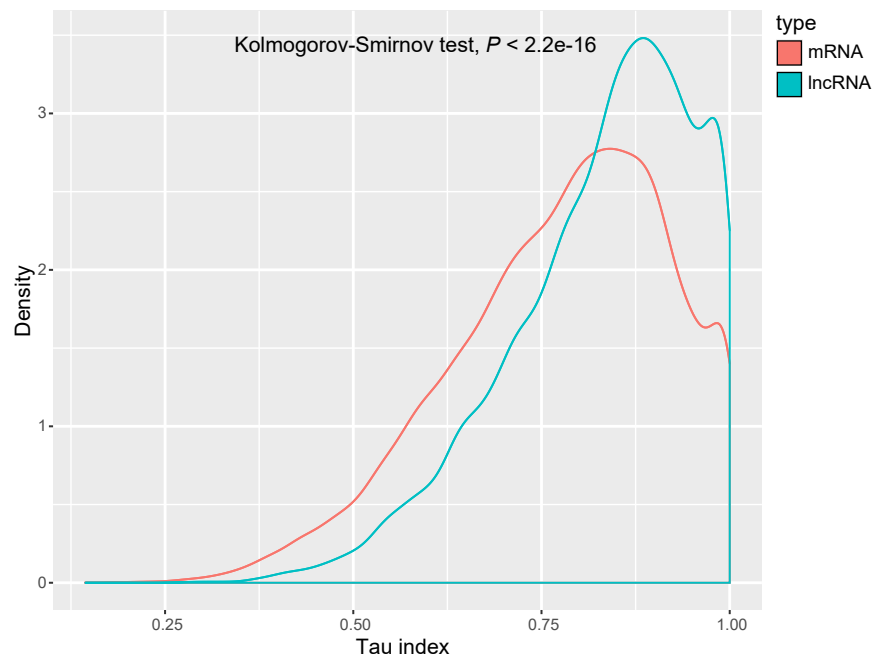

B

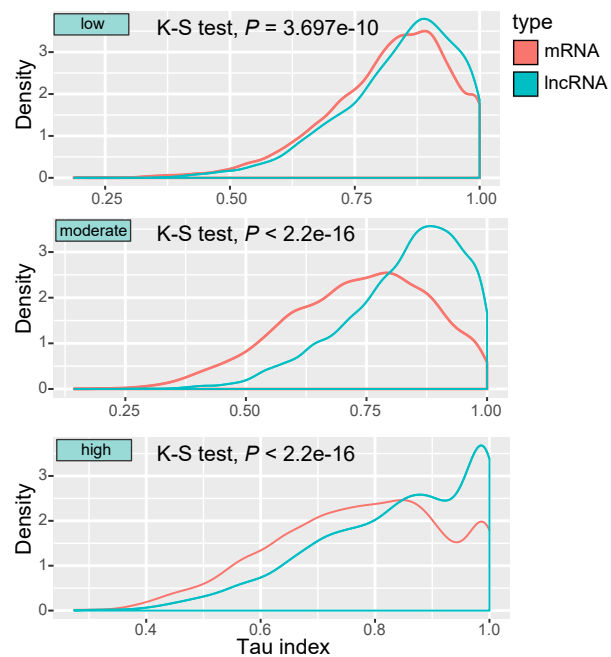

Supplement: Supplementary file 13 — Additional file 13: Figure S7. Density plot showing the distribution of tau index for PCGs and lncRNAs across nine tissue samples. (A) The distribution of tau index for all expressed PCGs and lncRNAs. (B) The distribution of tau index for PCGs and lncRNAs in three groups with different expression levels. Statistical significance of the difference in tau index between PCGs and lncRNAs was determined using the Kolmogorov-Smirnov test. [file 12864_2020_7313_MOESM13_ESM.pdf]

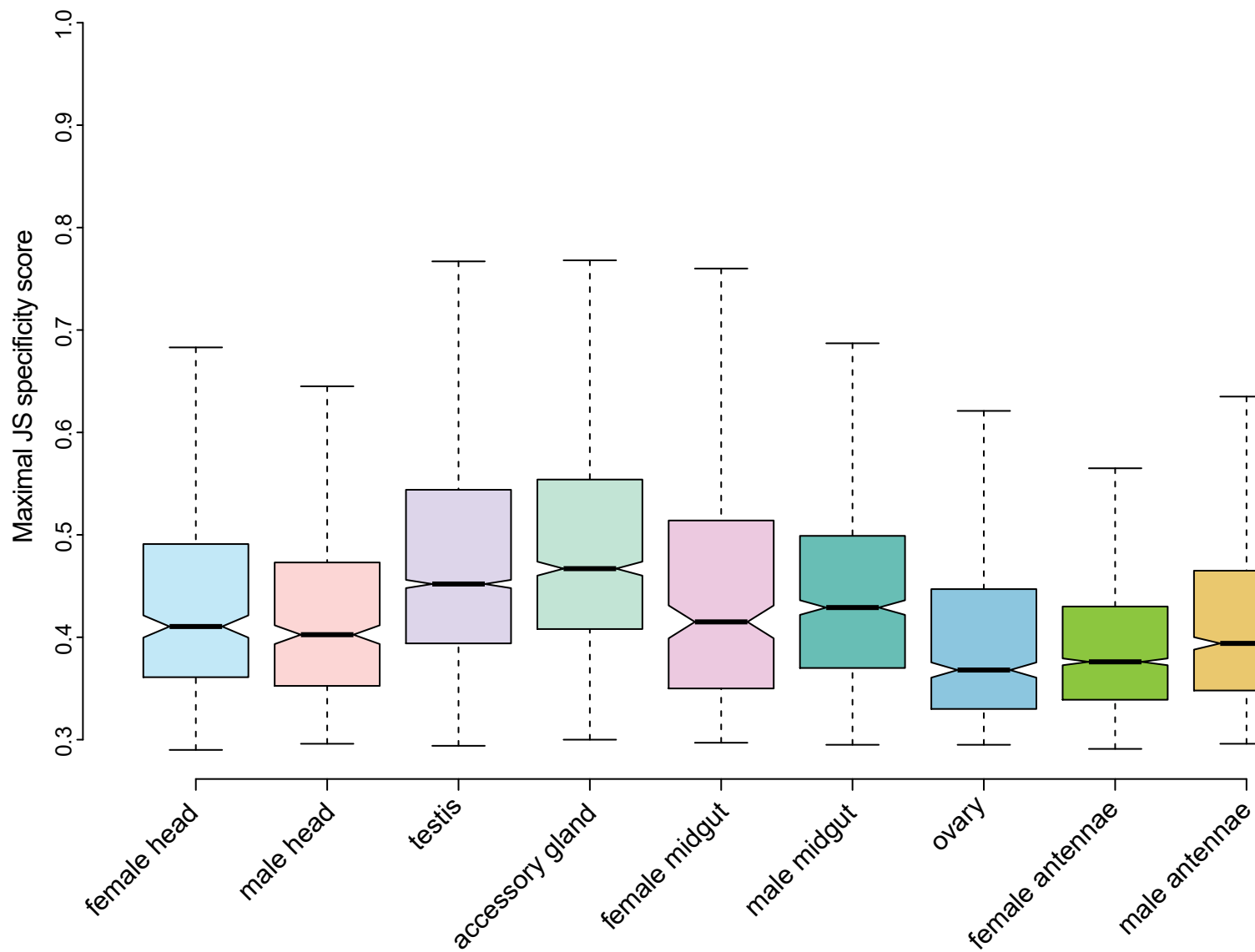

Supplement: Supplementary file 14 — Additional file 14: Figure S8. Distribution pattern of the JS specificity scores of tissue-specific genes in different tissues. [file 12864_2020_7313_MOESM14_ESM.pdf]

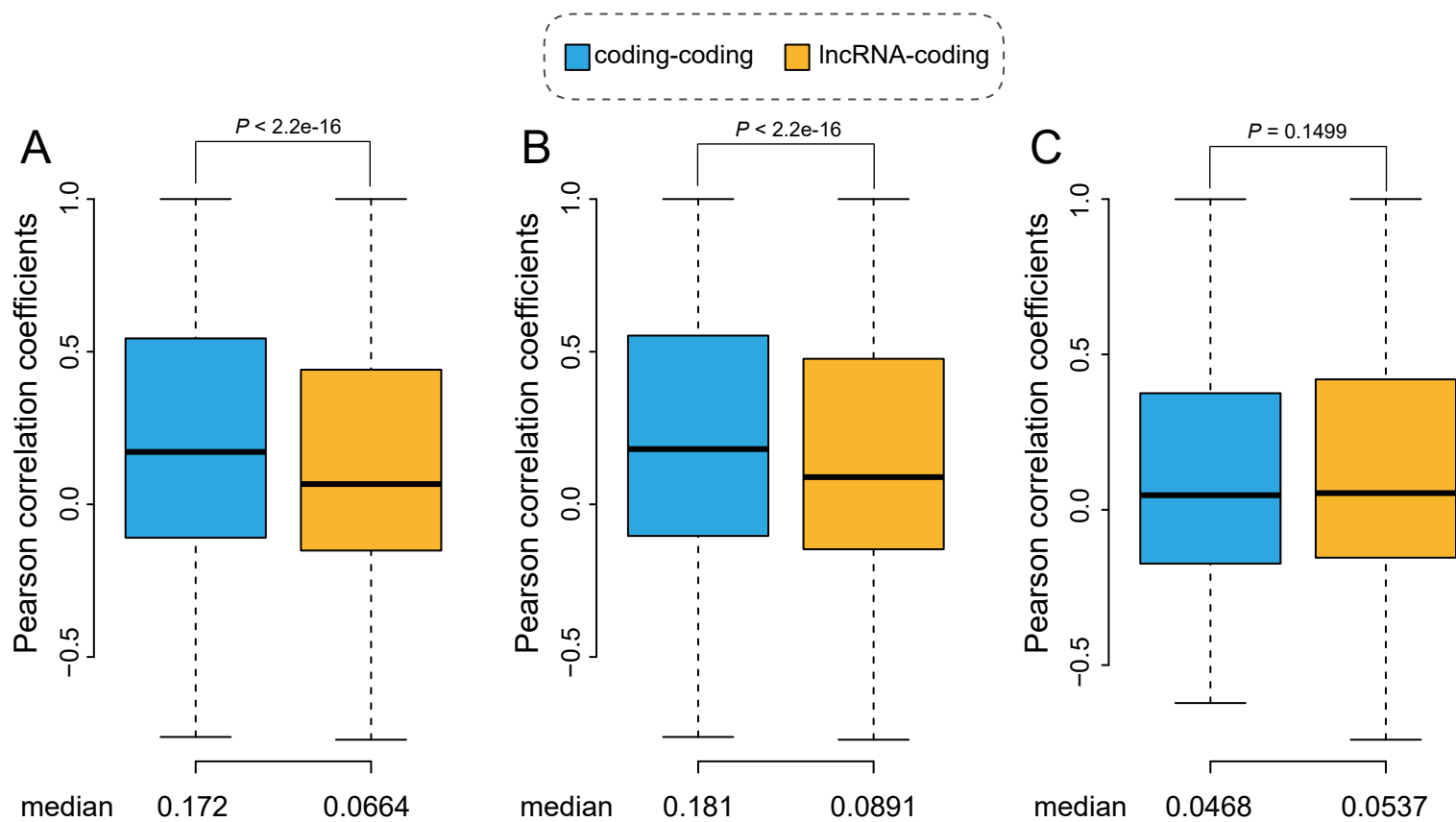

Supplement: Supplementary file 15 — Additional file 15: Figure S9. Distribution of PCCs for neighboring GPs. (A) Boxplot presentation of the distribution of PCCs for the entire set of neighboring gene pairs. (B) Boxplot showing the distribution of PCCs for non-overlapping gene pairs. (C) Boxplot representing the distribution of PCCs for overlapping gene pairs. [file 12864_2020_7313_MOESM15_ESM.pdf]

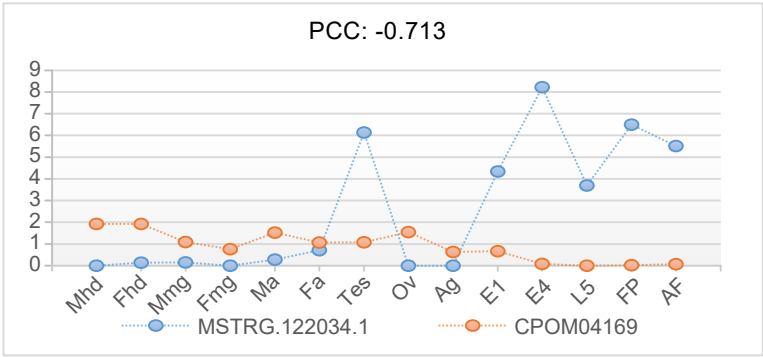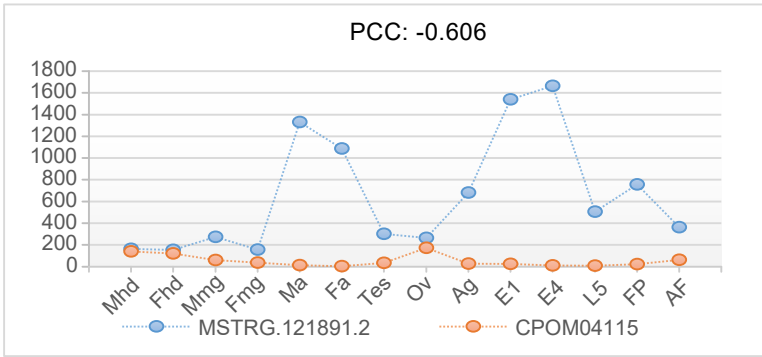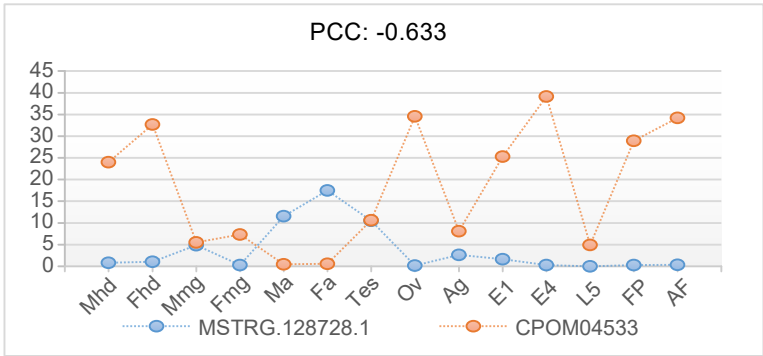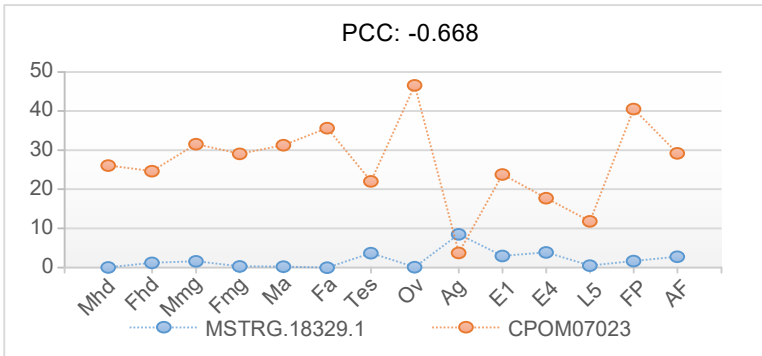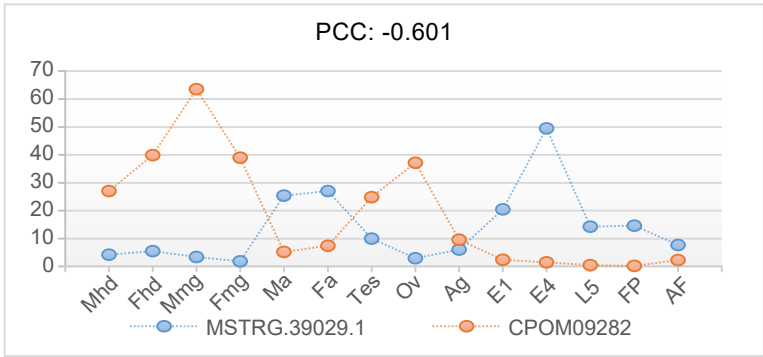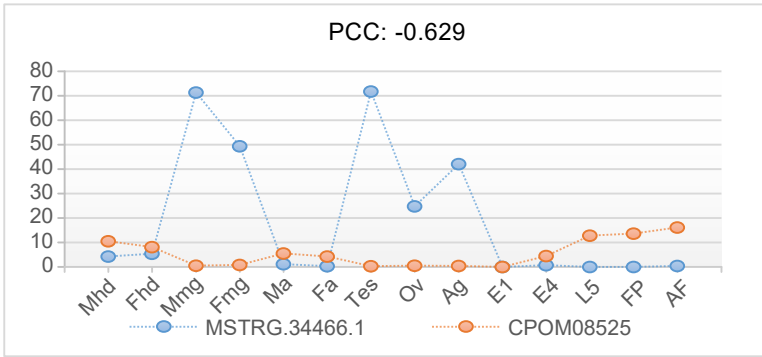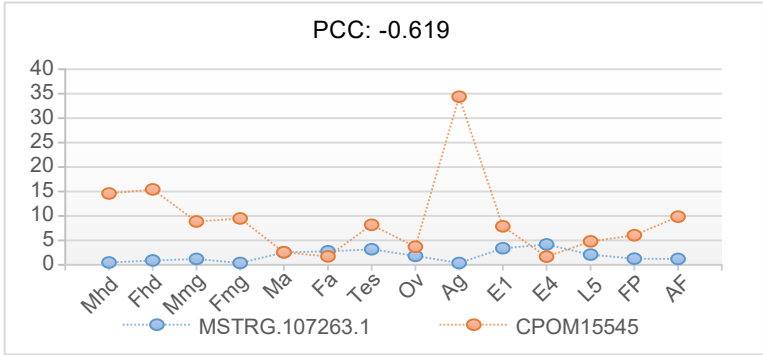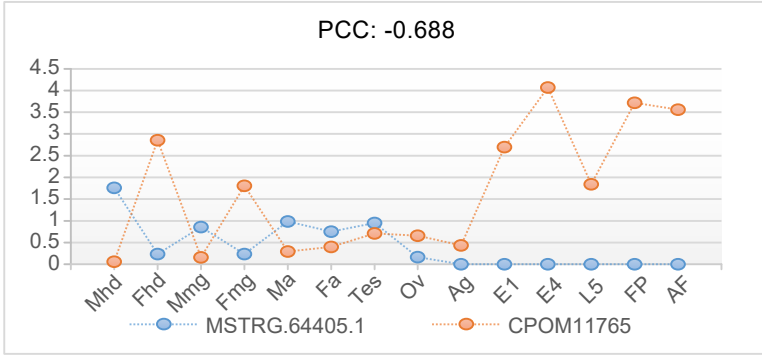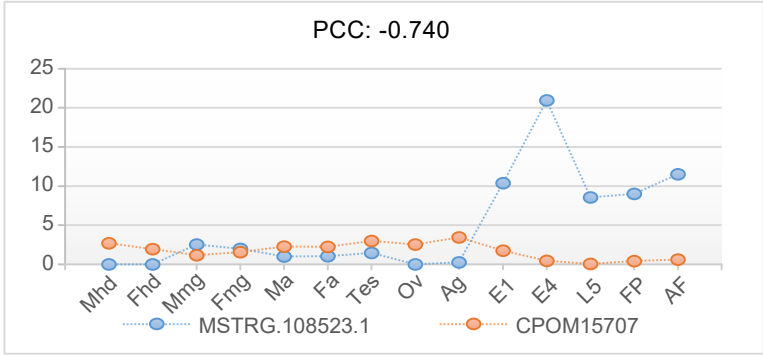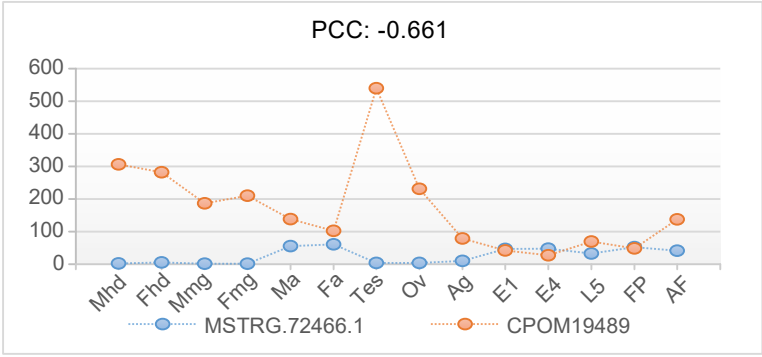

Supplement: Supplementary file 16 — Additional file 16: Figure S10. Ten representative overlapping lncRNA-coding GPs showing negative correlation with PCC < − 0.6. Blue circle represents lncRNA, and orange circle indicates PCG. Abbreviations are listed as follows: Mhd, male head; Fhd, female head; Mmg, male midgut; Fmg, female midgut; Ma, male antennae; Fa, female antennae; Tes, testis; Ov, ovary; Ag, accessory gland; E1, egg day 1; E4, egg day 4; L5, 5th-instar larva; FP, female pupa; AF, adult female. [file 12864_2020_7313_MOESM16_ESM.pdf]

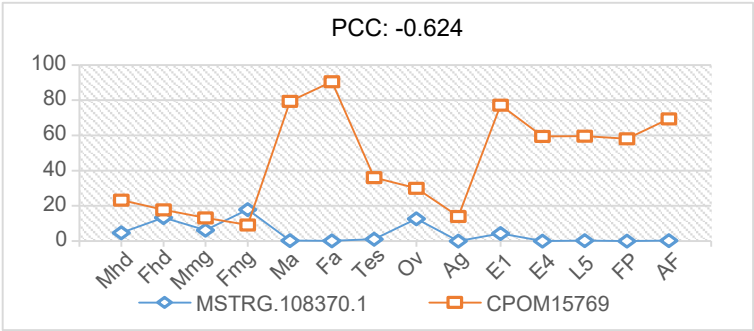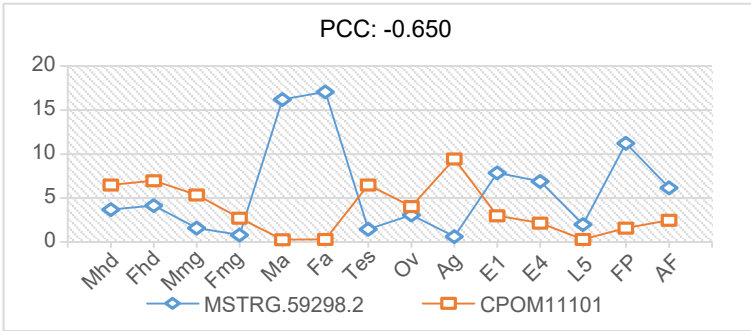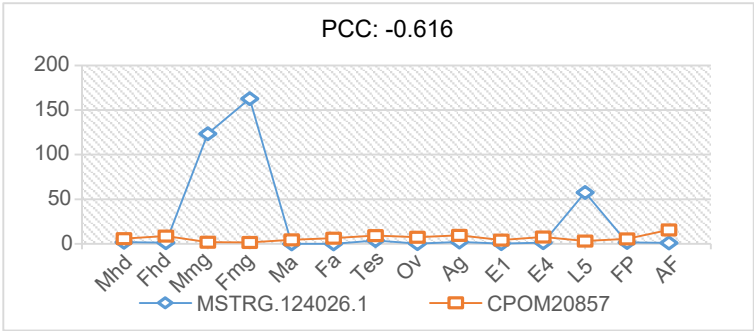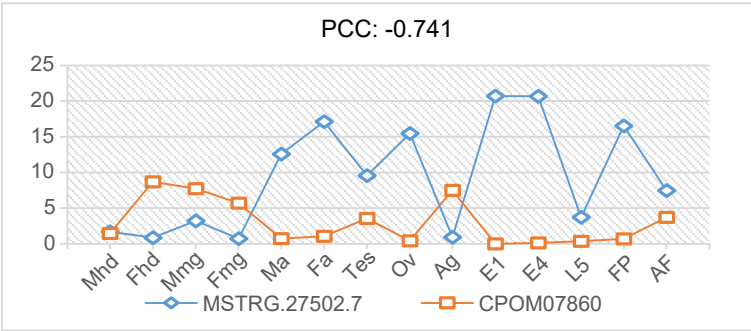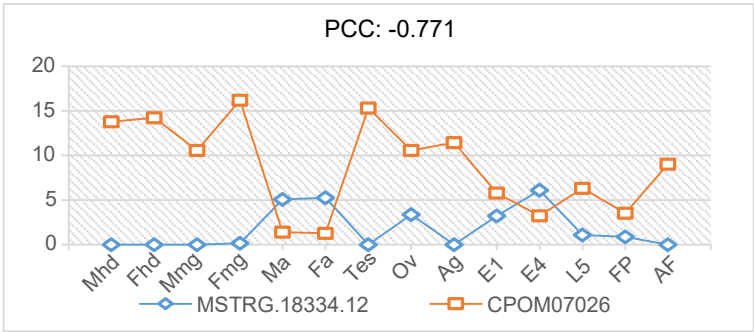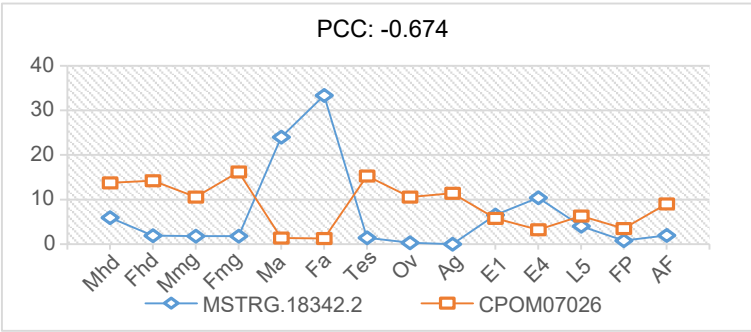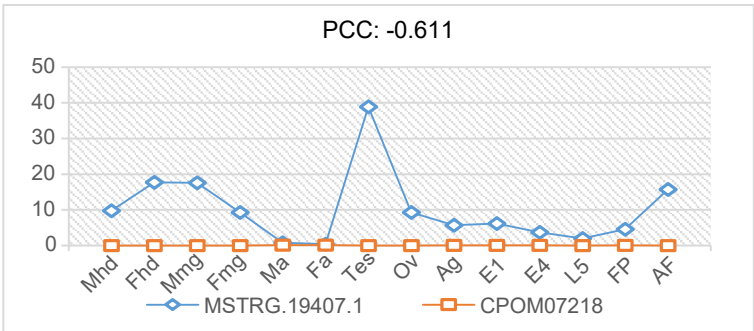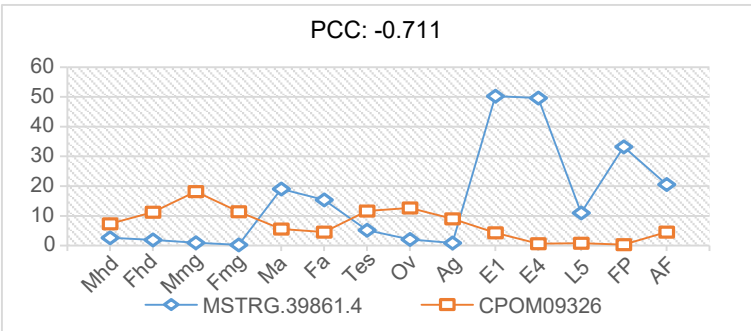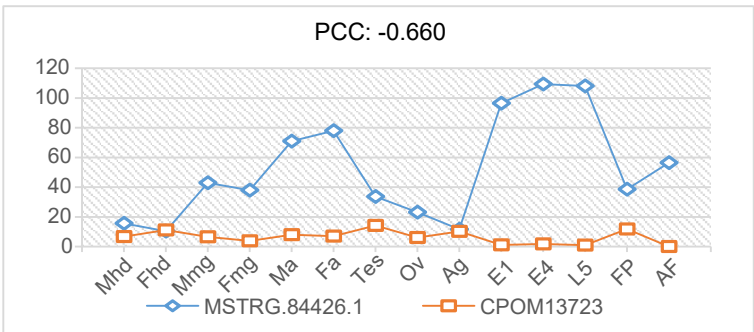

Supplement: Supplementary file 17 — Additional file 17: Figure S11. Nine representative non-overlapping lncRNA-coding GPs showing negative correlation with PCC < − 0.6. Blue diamond indicates lncRNA, and orange square represents PCG. Abbreviations are listed as follows: Mhd, male head; Fhd, female head; Mmg, male midgut; Fmg, female midgut; Ma, male antennae; Fa, female antennae; Tes, testis; Ov, ovary; Ag, accessory gland; E1, egg day 1; E4, egg day 4; L5, 5th-instar larva; FP, female pupa; AF, adult female. [file 12864_2020_7313_MOESM17_ESM.pdf]
